# Multi‐Organelle Stress‐Induced Paraptosis by a ROS‐Amplifying Nanocatalyst for Enhanced Cancer Immunotherapy

**DOI:** 10.1002/advs.202520031

**Published:** 2026-03-05

**Authors:** Zhe Yu, Haozhe Ren, Hua Li, Jinghan Zhao, Miaomiao Cao, Tian Tang, Youbei Qiao, Liting Chen, Tiehong Yang, Hong Wu

**Affiliations:** ^1^ Department of Pharmaceutical Analysis School of Pharmacy The Fourth Military Medical University Xi'an China; ^2^ School of Stomatology Lanzhou University Lanzhou China; ^3^ State Key Laboratory of Oral & Maxillofacial Reconstruction and Regeneration National Clinical Research Center for Oral Diseases Shaanxi Key Laboratory of Stomatology School of Stomatology The Fourth Military Medical University Xi'an China; ^4^ State Key Laboratory of Oral & Maxillofacial Reconstruction and Regeneration, National Clinical Research Center for Oral Diseases, Shaanxi Key Laboratory of Stomatology, School of Stomatology The Fourth Military Medical University Xi'an China; ^5^ Department of Chinese Materia Medica and Natural Medicines School of Pharmacy The Fourth Military Medical University Xi'an China; ^6^ Department of Radiation Oncology Xijing Hospital Air Force Medical University The Fourth Military Medical University Xi'an China

**Keywords:** autophagy, chemodynamic therapy, endoplasmic reticulum stress, immunogenic cell death, mitochondrial dysfunction, paraptosis

## Abstract

Chemodynamic therapy (CDT) is severely limited by inadequate intracellular H_2_O_2_ and frequent apoptosis resistance. Herein, a porous Fe_3_O_4_ nanoplatform is engineered through coating of Fe^3^^+^‐tannic acid complex onto nanoclusters simultaneously loaded with carbonyl cyanide 3‐chlorophenylhydrazone (CCCP, a mitochondrial uncoupler) and lactate oxidase (LOD). By self‐supplying H_2_O_2_ and concurrently blocking autophagic flux, this system is engineered to potentiate CDT efficacy. Beyond the expected therapeutic outcome, we uncover that this nanoplatform triggers an unconventional cell death pathway—paraptosis. Acid‐triggered dissociation in the tumor microenvironment releases Fe^2^^+^/Fe^3^^+^ for Fenton reactions while LOD simultaneously converts lactate to H_2_O_2_, overcoming the H_2_O_2_ bottleneck. CCCP further synergizes this process by collapsing the mitochondrial membrane potential, which amplifies ROS leakage and culminates in an oxidative storm. This dramatic surge in ROS directly induces mitochondrial dysfunction and initiates endoplasmic reticulum stress, while also suppressing autophagic flux. Collectively, these multi‐organelle stresses markedly exacerbate intracellular damage and lead to paraptotic cell death, characterized by extensive cytoplasmic vacuolization, eliciting robust immunogenic cell death. Combined with αPD‐L1, the nanoplatform potently activates antitumor immunity and suppresses both primary and distant tumors. This work pioneers a paraptosis activation strategy driven by an iron‐based nanodrug, redefining CDT efficacy through multi‐organelle stress synergy for amplified immunotherapy.

## Graphical abstract

The engineered TF‐Fe@LC nanoplatform programs paraptotic death via multipath ROS amplification that disrupts redox homeostasis, provoking mitochondrial dysfunction, autophagic blockade, and ER stress. This caspase‐independent process elicits robust immunogenic cell death, combining with αPD‐L1 to activate systemic CD8^+^ T‐cell immunity and suppress primary/abscopal tumors, advancing oxidative stress‐amplified immunotherapy.

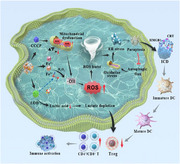

## 1 Introduction

Reactive oxygen species (ROS) play pivotal roles in maintaining cellular redox homeostasis [1, 2], regulating a broad spectrum of biological functions ranging from the meticulous regulation of signaling pathways and cellular differentiation to the orchestration of cellular damage response and ultimately, programmed cell death. A disruption of redox homeostasis can lead to cellular oxidative stress that gives rise to the pathogenesis of various malignancies, including cancer [3, 4]. Hydroxyl radicals (·OH) are the most active reactive oxygen species [5, 6], generating from the overexpressed H_2_O_2_ at tumor and catalytic materials through Fenton/Fenton‐like reactions [7, 8]. In this regard, as a Fenton‐based therapy, CDT has garnered significant attention over the last several decades, characterized by its specificity and non‐invasive nature [8, 9, 10, 11]. However, the efficacy of CDT is fundamentally constrained by insufficient intracellular H_2_O_2_ and frequent apoptosis resistance in tumors [12].

Enzyme‐mediated strategies (e.g., lactate oxidase, glucose oxidase) have been developed to generate H_2_O_2_ in situ from tumor metabolites [13, 14]. Meanwhile, overcoming apoptosis resistance requires the induction of alternative, non‐apoptotic cell death modalities. Paraptosis [15], a non‐apoptotic programmed cell death modality that is pathologically defined by cytoplasmic vacuolization originating from swollen endoplasmic reticulum (ER) and/or mitochondria (Mito), represents a compelling alternative. While the upstream signaling cascades triggering paraptosis require further elucidation, its occurrence is invariably linked with massive ROS burst [16], severe mitochondrial dysfunction [17], and endoplasmic reticulum stress (ERS) [18, 19]. Mounting evidence reveals an expanding repertoire of small‐molecule compounds capable of inducing paraptosis in cancer cells [20, 21, 22], highlighting its therapeutic promise in oncology. However, the deficient mitochondrial selectivity and suboptimal antitumor efficacy of such compounds constrained their therapeutic outcomes. To date, only a limited number of metal‐based nanodrugs have been reported to trigger paraptosis, primarily relying on Cu^2+^‐mediated cytotoxicity through toxic ligand coordination [23], calcium overload‐induced mitochondrial dysfunction via diverse Ca^2^^+^ modulators [24, 25], or the delivery of paraptosis‑inducing small molecules (e.g., morusin) [26, 27, 28]. Pioneering work further reveals Ni^2^^+^ as a paraptosis inducer [29], while iron‐mediated paraptosis induction remains unexplored.

In contrast to these strategies, which predominantly follow a “payload‐delivery” paradigm by administering known toxic inducers, we conceptualized a novel “metabolic reprogramming” approach. Our strategy leverages iron ions to initiate CDT, which cooperates with lactate depletion and mitochondrial disruption to trigger an intracellular cascade culminating in paraptosis. CCCP, a proton ionophore, eliminates the mitochondrial membrane potential and is routinely used as a mitophagy inducer [30]. It is demonstrated that CCCP induces ROS production in mitochondria and triggers ERS by disrupting ER protein homeostasis [31]. Notably, iron ions synergize with CCCP to dramatically boost ROS generation through mitochondrial dysfunction [32, 33], providing a rational basis for orchestrating dual organelle stress toward paraptosis.

**SCHEME 1:**
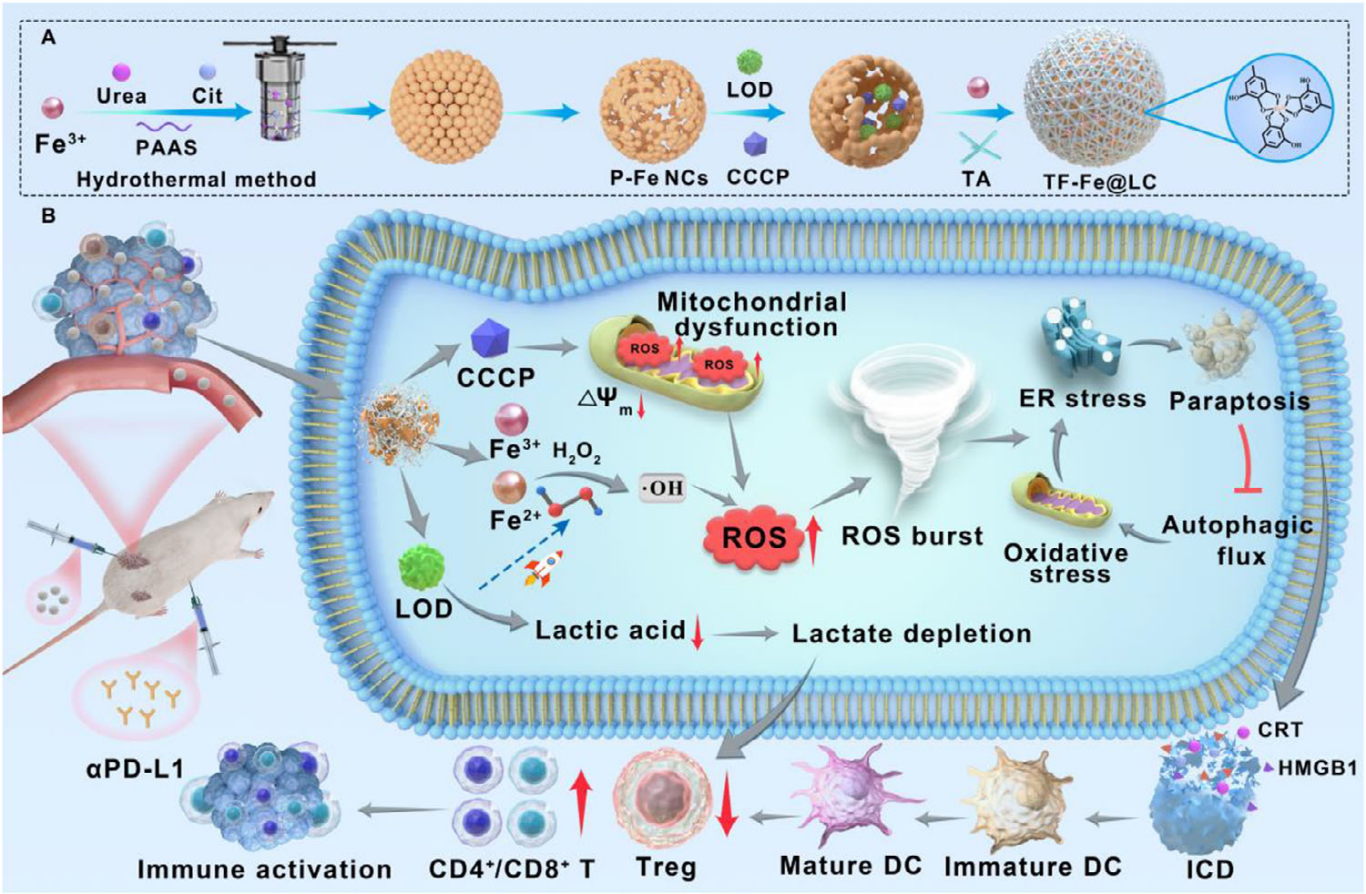
Schematic illustration of porous Fe_3_O_4_‐Engineered ROS avalanche and Mito‐ER chaos driving paraptosis‐immunotherapy synergy. (A) Synthesis process of TF‐Fe@LC. (B) Illustration of the mechanism of TF‐Fe@LC induced paraptosis‐like cell death through multi‐pronged amplification of ROS and activated immunity response through immunogenic cell death induction.

Herein, we construct a CDT‐amplified nanoplatform (TF‐Fe@LC) designed to co‐deliver CCCP and LOD using porous Fe_3_O_4_ nanoclusters (P‐Fe NCs), followed by coating with a pH‐responsive Fe^3^^+^‐tannic acid network (Scheme 1). Initially aimed at enhancing CDT and autophagic sensitization, this system is expected to self‐supply H_2_O_2_ (via LOD) and amplify oxidative stress through Fe‐mediated Fenton reactions and CCCP‐induced mitochondrial ROS leakage. Unexpectedly, beyond merely improving cytotoxic efficacy, we observe that TF‐Fe@LC elicits extensive cytoplasmic vacuolization and robust immunogenic cell death (ICD), hallmarks of paraptosis. Through transcriptomic analysis, Western blot, and cell imaging, we systematically demonstrate that this nanoplatform triggers paraptosis via synergistic mitochondrial dysfunction and ERS, mechanistically involving the PERK‐eIF2α‐ATF4‐CHOP axis and impaired autophagic flux. Finally, CDT‐driven paraptosis effectively evokes ICD, and its combination with αPD‐L1 significantly suppresses the proliferation of both primary and distant tumors. Herein, we not only report the fabrication of a self‐reinforcing nanoplatform for synergistic therapy but, more importantly, demonstrate for the first time that this system potently induces paraptosis in tumor cells. This discovery provides a new paradigm for nanomaterial‐based cancer therapy through the activation of alternative cell death pathways.

## 2 Results and Discussion

### 2.1 Synthesis and Characterization of TF‐Fe@LC

**FIGURE 1:**
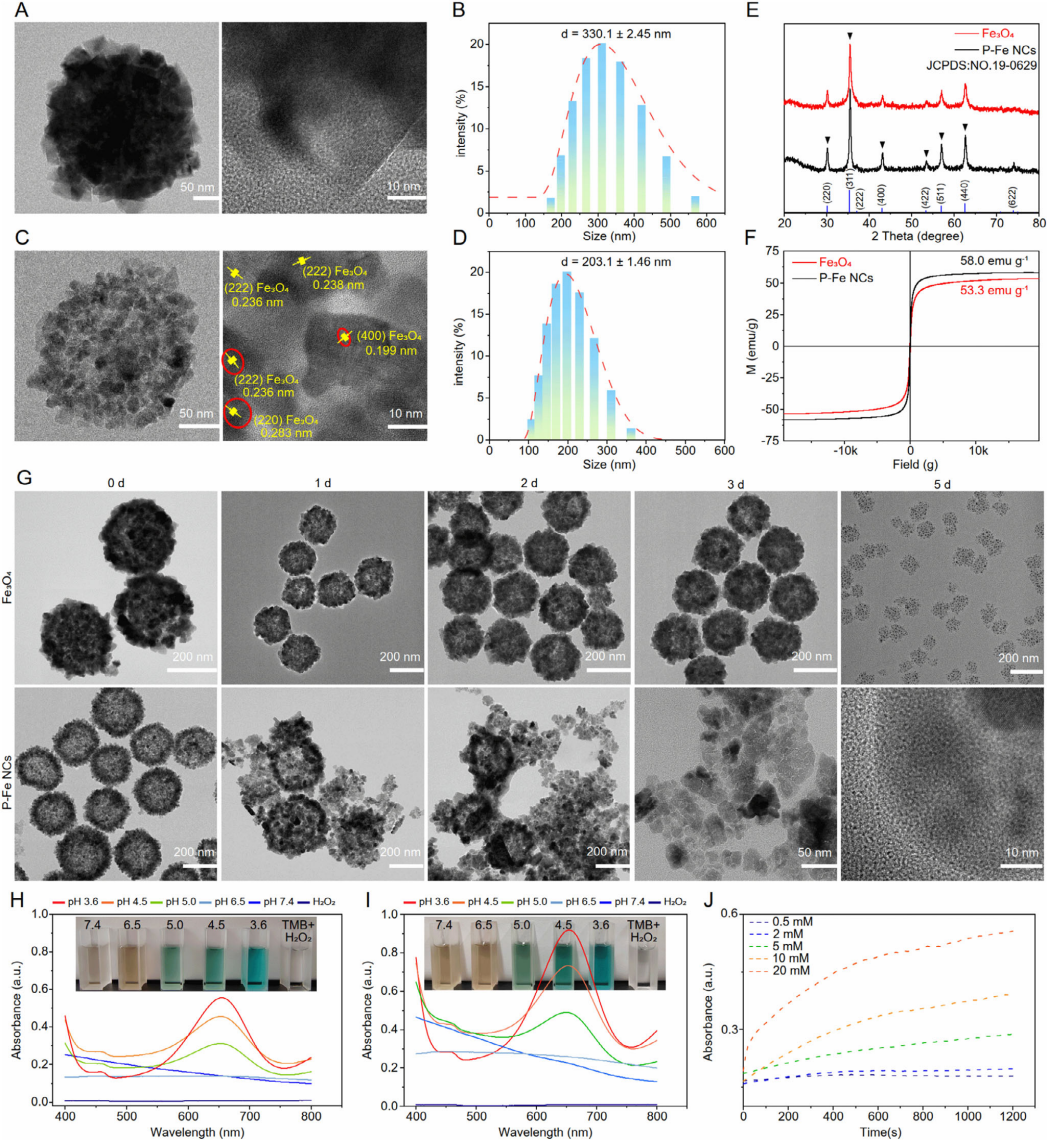
Characterization of P‐Fe NCs. TEM images of (A) Fe_3_O_4_ and (C) P‐Fe NCs; left: magnified TEM image of a single nanocluster, right: HRTEM images. Hydrodynamic size distribution of (B) Fe_3_O_4_ and (D) P‐Fe NCs. (E) XRD spectra. (F) Magnetic hysteresis curves. (G) TEM images of the structural evolution in PBS solution (pH 5.0) (upper: Fe_3_O_4_, lower: P‐Fe NCs). UV–vis spectra of TMB solutions catalyzed by (H) Fe_3_O_4_ before etching and (I) P‐Fe NCs at different pH. (J) Time‐course absorbance at 652 nm of oxTMB catalyzed by P‐Fe NCs upon the addition of various concentrations of H_2_O_2_ (0.5, 2, 5, 10, and 20 mm).

P‐Fe NCs was synthesized by a modified hydrothermal method with FeCl_3_ as the iron source and sodium polyacrylate as dispersant [34, 35]. The original synthesized Fe_3_O_4_ with a diameter of 300 nm were tightly aggregated by a number of nanocrystals (Figure 1A), amongst which smaller Fe_3_O_4_ nanocrystals could be selectively etched by citric acid. After a certain etching time, the P‐Fe NCs with a diameter of 180 nm were finally obtained (Figure 1C). As demonstrated by high‐resolution transmission electron microscopy (HRTEM), the nanocluster was composed of uniformly oriented ultrasmall nanocrystals (3–6 nm in size), with lattice fringe spacings matching the magnetite (Fe_3_O_4_). The hydrodynamic size of Fe_3_O_4_ before and after eaching were respectively 330 and 203 nm (Figure 1B,D). XRD analysis confirmed the typical crystalline structures of P‐Fe NCs, namely the (220), (311), (400), (422), (511) and (440) planes, which matched well with JCPDS 19–0629 (Figure 1E). The obtained P‐Fe NCs exhibited high water dispersibility, attributed to the carboxyl groups anchored on their surface, as confirmed by FTIR (Figure S1). Meanwhile, the saturation magnetization of P‐Fe NCs was slightly higher than that of unetched Fe_3_O_4_ microspheres, which might be ascribed to the synergistic magnetic coupling at the porous interfaces (Figure 1F). Brunauer–Emmett–Teller (BET) analysis showed an increased surface area and pore size of Fe_3_O_4_ after etching (Figure S2 and Table S1), which certified that the smaller nanocrystals were dissolved during the etching process. The P‐Fe NCs, characterized by their unique structure with a pore size of 16.9 nm, demonstrated a capacity for carrying LOD, the diameter of which was 10 nm [36]. Taking the above synthesized P‐Fe NCs as the nanoplatform and nano‐Fenton agent, the dissociation behavior in PBS solutions at pH 5.0 was investigated. Unlike the relatively inert structural evolution of the Fe_3_O_4_ before etching, TEM images displayed the spherical morphology of P‐Fe NCs that underwent deformation within 1 day (Figure 1G), transforming into irregular shapes, accompanied by the observation of smaller nanoclusters. 3 days later, the P‐Fe NCs totally dissociated into debris. After 5 days, the spherical nanoclusters completely disappeared and decomposed entirely into discrete nanocrystals. The iron release profile demonstrated pH‐dependent behavior, with minimal release observed at pH 7.4. In contrast, acidic conditions significantly enhanced ion dissolution, achieving 220 µm release for Fe ion within 48 h (Figure S3). These results demonstrated that P‐Fe NCs could rupture in the weak acidic environment and contributed to the release of Fe^2+^ to ensure ROS generation.

The catalytic activity of P‐Fe NCs was investigated by using TMB as the OH indicator. As illustrated in Figure 1H,I, both pristine and etched Fe_3_O_4_ exhibited pH‐dependent catalytic kinetics, with the P‐Fe NCs demonstrating superior peroxidase‐like activity. The enhanced catalytic performance originated from the synergistic structural advantages of shortened electron transfer distance between Fe^2^^+^/Fe^3^^+^ sites in the porous architecture and localized H_2_O_2_ enrichment within nanochannels that collectively accelerated Fenton reaction kinetics. Further, classical Michaelis–Menten kinetics were used to evaluate the catalytic performance of the P‐Fe NCs. The time‐course absorbance changes were recorded following the addition of TMB to solutions of P‐Fe NCs (25 µg/mL) in the presence of varying H_2_O_2_ concentrations (0.5, 2, 5, 10, and 20 mm) as substrates (Figure 1J). The Michaelis constant (K_M_) and maximum reaction velocity (V_max_) were calculated to be 0.395 mm and 6.25 × 10^−7^ Ms^−1^ for P‐Fe NCs (Figure S4), indicating high catalytic activity.

**FIGURE 2:**
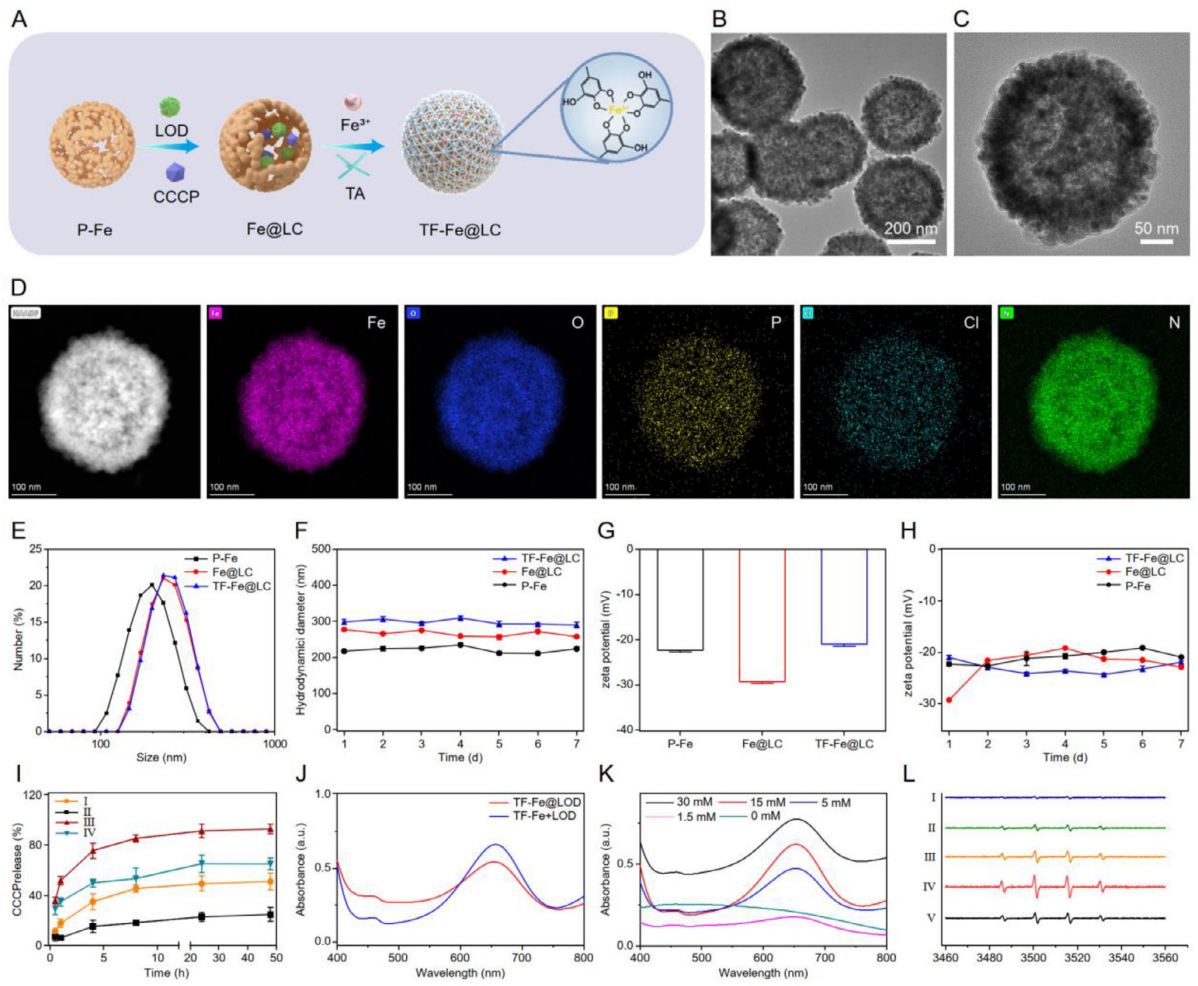
Preparation and characterization of TF‐Fe@LC. (A) Schematic illustration for the preparation of TF‐Fe@LC. (B,C) Representative TEM images of TF‐Fe@LC. (D) Elemental distribution mapping of TF‐Fe@LC. (E) Hydrodynamic sizes and (G) zeta potentials of P‐Fe, Fe@LC, and TF‐Fe@LC. (F) Size and (H) zeta potential stability, *n* = 3. (I) Time‐course of CCCP release from Fe@LC and TF‐Fe@LC at varying pH. I: TF‐Fe@LC (pH 5.0), II: TF‐Fe@LC (pH 6.5), III: Fe@LC (pH 5.0), IV: Fe@LC (pH 6.5), *n* = 3. (J) Absorbance at 652 nm of oxTMB catalyzed by TF‐Fe+LOD and TF‐Fe@LOD in the presence of lactic acid (12 mm). (K) Lactic acid concentration‐dependent oxTMB (652 nm) formation by TF‐Fe@LC‐mediated cascaded catalysis. (L) EPR spectra of TF‐Fe@LC with DMPO. I: +H_2_O_2_ (pH 7.4), II: +H_2_O_2_ (pH 6.5), III: +H_2_O_2_ (pH 5.0), IV: +H_2_O_2_ (pH 4.5), V: +LA (pH 5.0).

TF‐Fe@LC was first prepared by mixing LOD and CCCP with P‐Fe NCs. Subsequently, ferric chloride and TA were consecutively introduced to the prepared Fe@LC suspension, forming a cross‐linked thin film on its surface through the coordination between the abundant phenolic hydroxyl groups of TA and Fe^3+^ ions [37]. This process was fabricated in Figure 2A. TEM images showed a misty unilamellar membrane surrounding the TF‐Fe@LC NCs, while EDX elemental mapping confirmed the distribution of Fe, N, P, Cl, and O throughout the NCs. These results collectively verify the successful formation of an Fe^3+^‐TA (TF) shell and the effective loading of LOD and CCCP (Figure 2B–D). TF encapsulation resulted in an increased hydrodynamic diameter to 300 nm (Figure 2E) and elevated zeta potential following Fe^3^^+^ chelation, while maintaining an overall negative surface charge of −21 mV (Figure 2G). All nanosystems maintained stable hydrodynamic diameters and zeta potentials (Figure 2F,H), except for Fe@LC, which showed increased surface charge, potentially due to premature drug leakage. Further structural evidence was provided by FT‐IR analysis and XPS spectra. The infrared results indicated that the appearance of a new bond at 1202 cm^−^^1^ (Figure S5A), assigned to the phenolic C–O stretching vibration in the TA, confirmed the successful encapsulation of TF shell [38]. The XPS data confirmed the presence of Fe, C, and O in the TF‐Fe@LC (Figure S5B). In addition, high‐resolution spectra for TF‐Fe@LC were presented in Figure S5C–E. Deconvolution of the C 1s spectrum (Figure S5E) revealed three distinct components at binding energies of 284.8, 286.5, and 288.9 eV, corresponding to C–C, C–O, and O–C = O species, respectively. The latter two components are characteristic of TA, consistent with previous reports [38]. In the O 1s spectrum (Figure S5D), the peak at 530.7 eV was attributed to metal‐coordinated oxygen (Fe–O–C), while the component at 533.3 eV originated primarily from the C–O groups of the phenolic hydroxyl groups in TA. The Fe 2p spectrum (Figure S5C) displayed two main peaks at 710.5 eV (Fe 2p_3_/_2_) and 723.9 eV (Fe 2p_1_/_2_), confirming the presence of iron and indicating that the TA‐Fe coating forms a thin, conformal layer on the Fe_3_O_4_ surface. The drug loading efficiencies in TF‐Fe@LC were calculated by a subtraction method with 7.62% for LOD and 10.34% for CCCP. Specifically, LOD was determined by measuring eluted protein content using a BCA protein assay kit (Figure S6A,B), while CCCP was quantified by its standard curve at 370 nm (Figure S6C,D). Previous studies have well characterized the pH‐dependent decomposition of TA‐Fe complexes [37, 39, 40]. To quantitatively validate the pH‑responsive drug release profile of the nanoplatform, detailed kinetic studies were performed. The cumulative release of both CCCP and LOD at tumor‐relevant acidic pH conditions (pH 5.0, 6.5) was systematically investigated. As shown in Figure 2I, rapid and pH‑modulated CCCP release in Fe@LC was observed, with near‑complete release (>90%) within 24 h at pH  5.0. A discernible acceleration was observed under more acidic conditions, attributable to the gradual disintegration of the P‐Fe NCs. This confirms the absence of a gatekeeping effect but indicates the acid‑sensitivity of the inorganic carrier itself. In contrast, TF‑Fe@LC exhibited a much more pronounced and controlled pH‑responsive release kinetics for both payloads (Figure 2I; Figure S7), owing to the additional acid‑labile TF coordination shell. At tumor‑stroma pH 6.5, release was minimal, with only ≈22.8 % of CCCP and ≈16.8 % of LOD released after 24 h. Further acidification to lysosomal pH 5.0 triggered significantly accelerated release, with cumulative amounts attaining ≈49.3 % for CCCP and ≈39.0 % for LOD within the same period. The dissociation of TF coating at pH 5.0 progressively accelerated drug release, resulting in significantly higher release rates compared to pH 6.5 conditions.

It has been well‐established that tumor cells predominantly rely on anaerobic glycolysis (Warburg effect) [41], resulting in the accumulation of lactic acid (LA, 10–30 mm) in the TME [37]. TF‐Fe@LC catalyzed the glycolytic byproduct lactate into H_2_O_2_, which was subsequently catalyzed by the P‐Fe NCs into ·OH via Fenton reaction. The generation of H_2_O_2_ catalyzed by LOD was quantitatively assessed across a range of lactate concentrations and under different pH conditions (Figure S8). The cascade catalytic performance was further investigated, the physical mixture of TF‐Fe_3_O_4_ and LOD demonstrated stronger catalytic activity than the TF‐Fe@LOD composite (Figure 2J). As shown in Figure 2K, the UV–vis absorbance of TMB solution catalyzed by TF‐Fe@LC exhibited a lactate concentration‐dependent catalytic profile, highlighting its lactate‐responsive catalytic behavior. The cascade catalysis of TF‐Fe@LC was also confirmed by electron paramagnetic resonance (EPR) spectroscopy using 5, 5‐dimethyl‐1‐pyrroline‐N‐oxide (DMPO) as the OH‐trapping agent (Figure 2L).

### 2.2 Antitumor Effect of TF‐Fe@LC In Vitro

**FIGURE 3:**
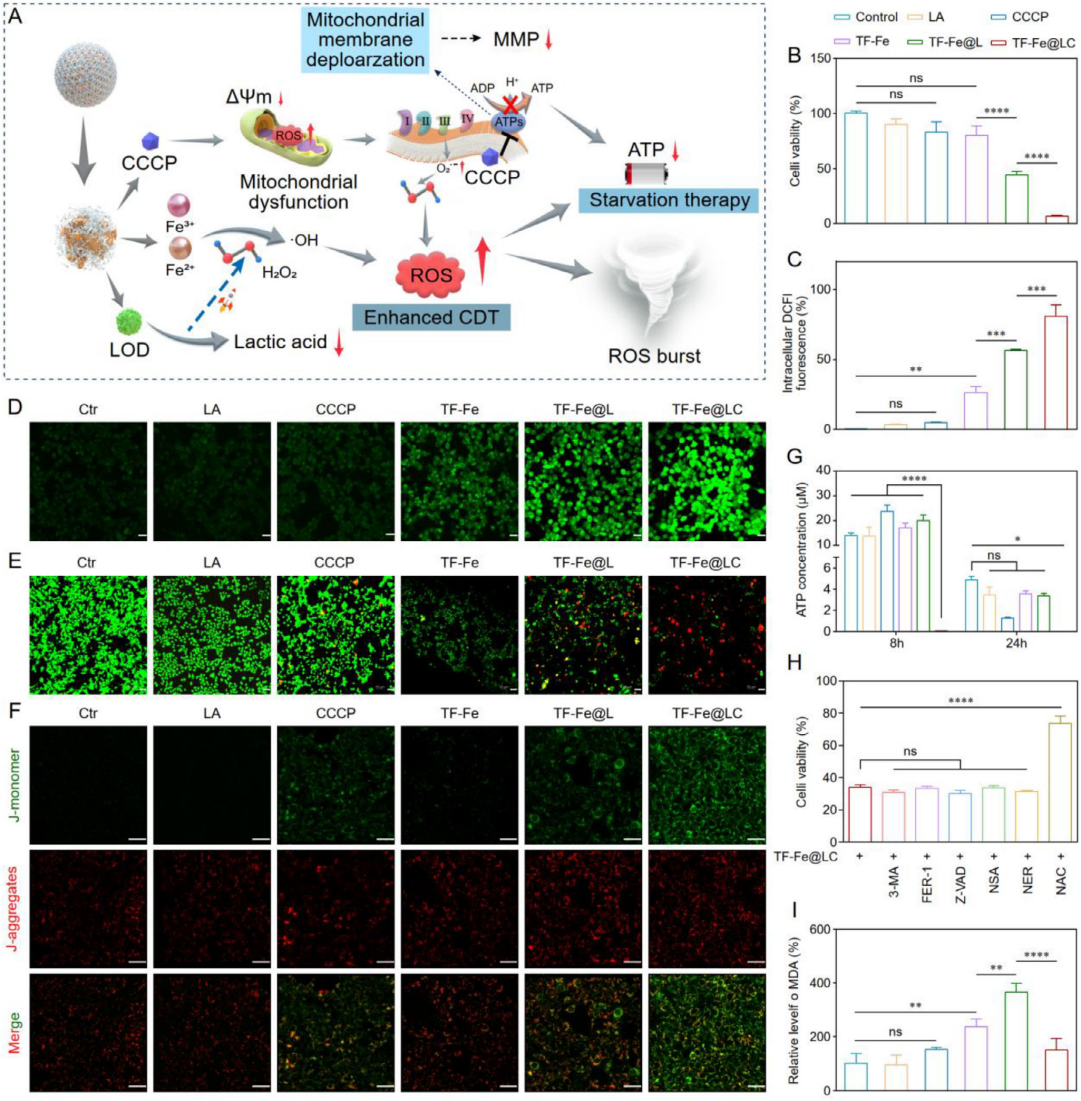
ROS‐triggered mitochondrial oxidative stress cascade and resulting cellular dysfunction in vitro. (A) Schematic illustration of TF‐Fe@LC‐induced programmed cell death via ROS burst and mitochondrial dysfunction. (B) Cell viabilities of 4T1 cells after different treatments, *n* = 6. (C) Quantitative detection of intracellular ROS generation after different treatments within 4T1 cells by flow cytometry, *n* = 3. (D) CLSM observation of intracellular ROS after different treatments. Scale bar: 20 µm. (E) CLSM images of 4T1 cells after different treatments stained with Calcein AM and PI. Green fluorescence: live cells, red fluorescence: dead cells. Scale bar: 50 µm. (F) CLSM images of TF‐Fe@LC‐induced mitochondrial depolarization. Green fluorescence: Monomers, red fluorescence: Aggregates. Scale bar: 50 µm. (G) Relative ATP levels of 4T1 cells after different treatments for 8 h or 24 h, *n* = 3. (H) Relative cell viabilities of 4T1 cells following TF‐Fe@LC treatment with different cell death inhibitors, *n* = 6. (I) Relative MDA level of 4T1 cells after different treatments, *n* = 3. ^*^p < 0.05, ^*^ ^*^p < 0.01, ^**^ ^*^p < 0.001, ^***^ ^*^p < 0.0001.

The TF coating increased the hydrophilicity, while its potential impact on cellular uptake prompted detailed investigation. As shown in Figure S9, RhB‐labeled TF‐Fe@LC exhibited delayed cellular uptake compared to Fe@LC. Red fluorescence (RhB signal) appeared only after 2 h of incubation and reached maximum intensity at 4 h, indicating the hydrophilic TF coating slowed but did not prevent cellular internalization. After effective cellular internalization, the acidity‐responsive TF‐Fe@LC rapidly disintegrated in the TME to release Fe^2+^/Fe^3+^, CCCP, and LOD. The liberated Fe ions induced Fenton reactions by generating ⋅OH, and LOD catalyzed lactate into H_2_O_2_ and pyruvate, establishing a self‐sustaining catalytic cascade in situ. The released CCCP exacerbated oxidative stress in cells through mitochondrial dysfunction, thereby creating a self‐reinforcing catalytic loop that intensifies ROS generation. As shown in Figure 3A, this triad of effects, consisting of Fenton catalysis, lactate‐driven H_2_O_2_ regeneration, and mitochondrial dysfunction, formed a closed‐loop system that perpetuated ROS accumulation while crippling cellular energy metabolism, ultimately leading to cell death. Initially, the antitumor effects of TF‐Fe@LC were evaluated on 4T1 cells via CCK 8 assay and live/dead co‐staining assay. 4T1 cells were divided and treated with control (ctr), lactate (LA), CCCP (C), TF‐Fe, TF‐Fe@L, and TF‐Fe@LC, respectively. According to Figure S10, TF‐Fe with/without H_2_O_2_ shown minimal inhibitory effect on 4T1 cells under neutral conditions. Hence, 12.0 mm lactate was supplemented into RPMI‐1640 culture medium to adjust the pH to 6.0. From Figure 3B and Figure S11, TF‑Fe@L and TF‑Fe@C treatment for 24 h resulted in 4T1 cell viability rates of 52.9% and 64.1% at 50 µg/mL of Fe_3_O_4_, respectively. In contrast, the TF‑Fe@LC group showed a markedly reduced cell viability of only 12.4%. To evaluate the synergistic effect of different treatment modalities, we calculated the combination index (CI), where a CI value < 1 indicates synergy. The calculated CI value for TF‑Fe@L and TF‑Fe@C was 0.366, which is well below 1. This result clearly demonstrates that TF‑Fe@LC exerts a significant synergistic antitumor effect through the coordinated action of mitochondrial oxidative stress and the catalytic cascade. To directly verify the availability of the cascade enzymatic substrate and the catalytic function of TF‐Fe@LC, the intracellular lactate concentration post‐treatment was measured. As shown in Figure S12, TF‐Fe@LC treatment led to a significant reduction in intracellular lactate levels compared to the control and other treatment groups. After 12 h, the lactate content in the TF‐Fe@LC group was measured at 47.7% ± 3.44% of the control level, confirming efficient lactate consumption by the nanozyme. This result confirms that intracellular lactate is sufficient to sustain the continuous catalytic activity of TF‐Fe@LC, providing the necessary fuel for the subsequent cascade. Consistent with the CCK‐8 assay results, Calcein AM/PI co‐staining demonstrated substantial red fluorescence (dead cells) and rare green fluorescence (live cells) in TF‐Fe@LC‐treated groups compared to other groups (Figure 3E). To investigate that TF‐Fe@LC primarily induced tumor cell death through triggering a ROS burst storm, a series of in vitro assays were performed. The ROS production within 4T1 cells were detected by the fluorescence probe 2',7'‐dichlorofluorescein diacetate (DCFH‐DA). Figure 3C,D demonstrated the CCCP group exhibited only faint green fluorescence, indicating inadequate ROS induction by low‐dose mitochondrial uncoupling alone. Compared with TF‐Fe@L, TF‐Fe@LC generated significantly intensified fluorescence signals, confirming its superior capacity for ∙OH production through mitochondrial oxidative stress and catalytic cascade. High ∙OH levels reciprocally induced severe oxidative stress, resulting in mitochondrial dysfunction, which was assessed by using fluorescent probe JC‐1. As depicted in Figure 3F, a slight enhancement of green fluorescence (mitochondrial damage) was found in the CCCP group owing to the uncoupling of the mitochondrial electron transport chain. The significant green fluorescence of JC‐1 monomers was detected in TF‐Fe@LC treated group (Figure S13), which implied the maximum mitochondrial injury owing to the self‐amplifying ROS‐mitochondrial damage cycle. Since mitochondrial is the primary source of cellular ATP, quantification of ATP levels serves as a reliable indicator of mitochondrial functional integrity. After 8 h treatment, the CCCP group maintained ATP levels comparable to untreated control, whereas TF‐Fe@LC treatment induced severe ATP depletion, indicating complete energy exhaustion due to mitochondrial dysfunction (Figure 3G). After 24 h, all experimental groups exhibited reduced ATP levels compared to the 8 h timepoint due to confluent cell growth and nutrient depletion in the culture medium. Oxidative stress provokes widespread intracellular damage across multiple organelles. Malondialdehyde (MDA), a terminal lipid peroxidation biomarker, was also elevated in TF‐Fe@LC‐treated 4T1 cells (Figure 3I). Intriguingly, while TF‐Fe and TF‐Fe@L treatments induced substantial MDA accumulation (indicating severe lipid peroxidation), the TF‐Fe@LC group exhibited markedly reduced MDA levels (*p* > 0.05 vs control). Moreover, the cytotoxicity of TF‐Fe@LC was not affected by co‐incubation with the ferroptosis inhibitor ferrostatin‐1 (FER‐1). Other cell death inhibitors also showed no protective effects, but N‐acetylcysteine (NAC) significantly attenuated TF‐Fe@LC‐induced cytotoxicity (Figure 3H), confirming TF‐Fe@LC triggers ROS‐dependent cell death through non‐canonical pathways.

### 2.3 Mechanistically Interrogating the Paraptosis‐inducing Antitumor Effect Mediated by TF‐Fe@LC

**FIGURE 4:**
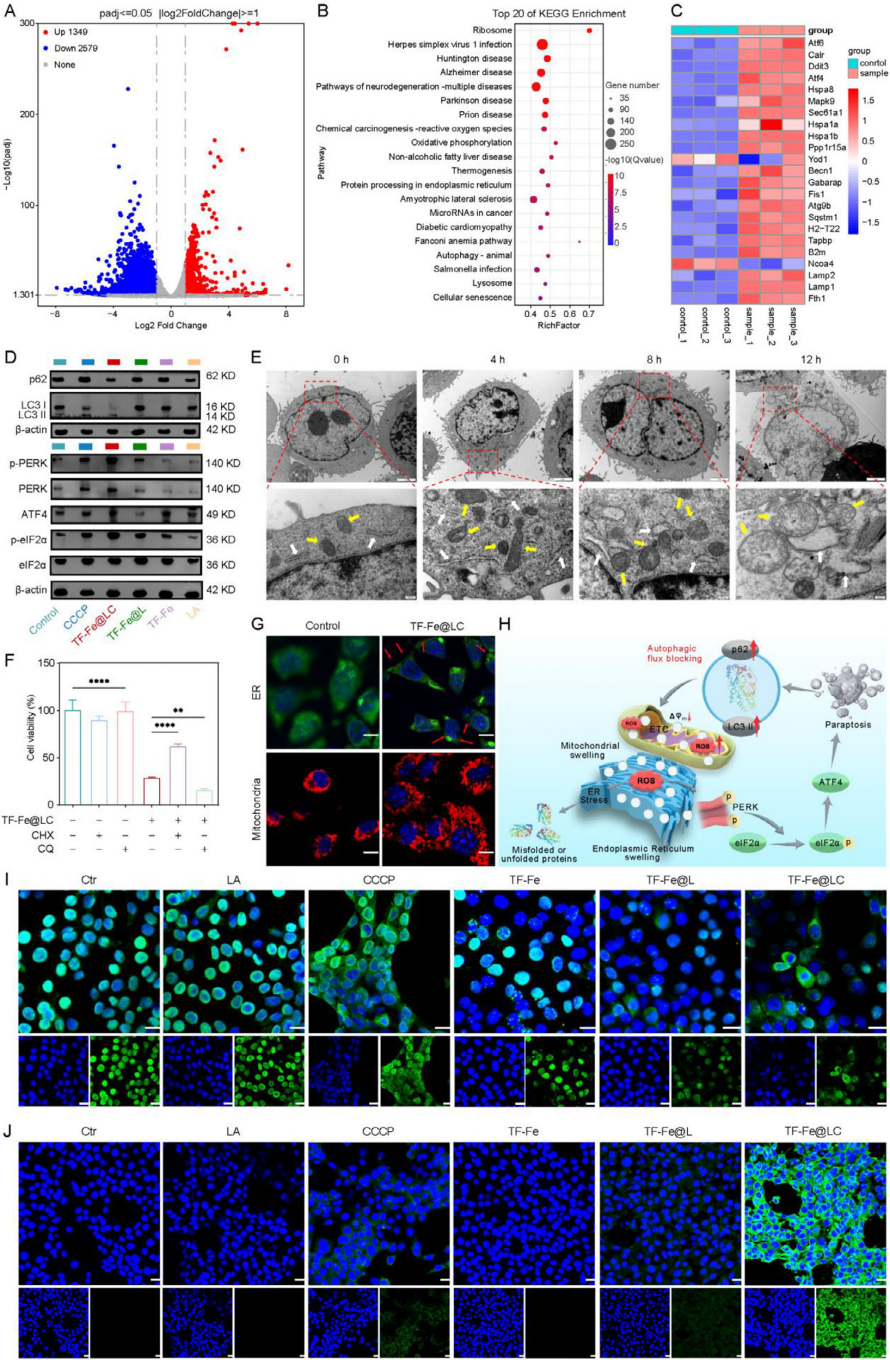
Analysis of TF‐Fe@LC‐induced paraptotic cell death. (A) Volcano plot analysis of TF‐Fe@LC‐induced transcriptional changes. (B) Bubble plot of the top 20 enriched KEGG pathways. (C) Heatmap of the related genes involved in ERS, mitophagy, and immune pathways. (D) Western blot of the autophagy and ERS‐related proteins in 4T1 cells. (E) Bio‐TEM images of TF‐Fe@LC‐induced cytoplasmic vacuolation in 4T1 cells over time (upper scale bar: 2 µm, lower scale bar: 200 nm). Swollen organelles: Mito (yellow arrows); ER (white arrows). (F) Effects of TF‐Fe@LC combined with CHX or CQ on 4T1 cell viability, *n* = 6. (G) CLSM images of 4T1 cells following TF‐Fe@LC treatment stained with ER‐tracker Green or Mito‐tracker Red. Scale bar: 10 µm. (H) The process of paraptosis through the PERK‐p‐eIF2α‐ATF4 pathway induced by TF‐Fe@LC. CLSM images of (I) HMGB1 release and (J) CRT expression in 4T1 cells after treatment with TF‐Fe@LC. Scale bar: 20 µm. ^**^p < 0.01, ^****^p < 0.0001.

Given the intriguing bioactivity of TF‐Fe@LC, RNA sequencing analysis was implemented to elucidate its underlying therapeutic mechanisms. As shown in the volcano plot (Figure 4A), a total of 3928 differentially expressed genes (DEGs) were identified between the TF‐Fe@LC‐treated group and the control group, including 1349 upregulated and 2579 downregulated genes. Kyoto Encyclopedia of Genes and Genomes (KEGG) and Gene Ontology (GO) enrichment analysis revealed overlapping pathways, including reactive oxygen species, oxidative phosphorylation, protein processing in the ER, lysosome, and autophagy regulation (Figure 4B; Figure S14). Notably, GO terms associated with antigen processing and presentation and vacuolar formation were also enriched, implying immune activation. Key upregulated genes included those involved in endoplasmic reticulum stress (ERS) (e.g., Atf4, Ddit3, Hspa5), mitophagy (e.g., Becn1, Sqstm1, Lamp2), and antigen presentation (e.g., B2m, Tapbp), consistent with pathway activation (Figure 4C). These findings were further corroborated by gene set enrichment analysis (GSEA) (Figure S15). Collectively, these data suggested the regulatory effects of TF‐Fe@LC on key proteins involved in autophagy and ERS, culminating in tumor cell eradication.

The mechanism by which TF‐Fe@LC induced cell death via autophagy and ERS was further investigated. Western blot analysis showed that TF‐Fe@LC treatment led to an elevated LC3‐II/I ratio and sustained levels of p62, ATG5, and Beclin1, suggesting that autophagy was not activated but rather that autophagic flux was blocked, owing to mitochondrial dysfunction and oxidative stress (Figure 4D; Figure S16). Additionally, the expression of BiP was significantly up‐regulated (Figure S17), confirming ERS induction [42, 43]. The PERK–eIF2α–ATF4 branch of the unfolded protein response was notably activated, consistent with its sensitivity to redox imbalance. Upon BiP dissociation, PERK was significantly upregulated and phosphorylated its substrate eIF2α, thereby suppressing global protein synthesis to alleviate ERS burden. However, phosphorylation of eIF2α paradoxically enhanced the translation of ATF4, thereby reprogramming the cellular redox signalling pathway [44]. Moreover, the oxidation‐induced damages in 4T1 cells could be markedly reversed with cycloheximide (CHX, a protein synthesis inhibitor) and aggravated with chloroquine (CQ, an autophagy flux inhibitor). The results indicated severe ERS and autophagy blockade, rather than other mechanisms, dominating the TF‐Fe@LC‐induced cell death (Figure 4F).

Time‐dependent Bio‐TEM analysis showed extensive cytoplasmic vacuolation derived from the dilation of ER and Mito in TF‐Fe@LC‐treated cells, which were morphological hallmarks of paraptosis (Figure 4E) [17, 29, 45]. The autolysosomes observed in Figure S18 indicated the accumulation of impaired autophagic flux. Furthermore, the treated cells were stained with ER‐Tracker Green or Mito‐Tracker Red to observe the vacuolization of ER or Mito (Figure 4G). Collectively, TF‐Fe@LC triggered ROS burst and mitochondrial dysfunction via cascade catalysis and mitochondrial electron decoupling. Concurrently, it exacerbated redox stress and activated the PERK‐p‐eIF2α‐ATF4 signaling axis, culminating in paraptosis with characteristic cytoplasmic vacuolization. Critically, this paraptosis disrupted CCCP‐induced mitophagy through blocking autophagic flux, establishing a ROS‐driven feedforward loop that amplified oxidative damage (Figure 4H).

Mitochondria‐associated ERS (MAERS) has been identified as an innovative approach for ICD induction [43, 46]. Thus, MAERS‐driven paraptosis as an ICD modality was assessed through hallmark ICD markers: calreticulin (CRT) and high mobility group box‐1 (HMGB1). The CLSM images demonstrated substantial HMGB1 release from nuclei (Figure 4I) and significant CRT exposure on the cell membrane (Figure 4J) in the TF‐Fe@LC group. To functionally validate that these damage‐associated molecular patterns (DAMPs) could initiate an immune response, we evaluated the maturation of dendritic cells (DCs), a critical downstream event of ICD. DCs were co‑cultured with conditioned medium from TF‑Fe@LC‑treated 4T1 cells. Flow cytometric analysis revealed a significantly higher percentage of CD11c^+^CD80^+^ and CD11c^+^CD86^+^ mature DCs after exposure to the TF‑Fe@LC‑conditioned medium compared to the control group (Figure S19). These results demonstrated that TF‐Fe@LC‐mediated robust ROS generation drove paraptotic cell death, thereby evoking amplified ICD via extracellular release of DAMPs, which in turn promoted DC maturation.

### 2.4 Biosafety and Antitumor Efficacy In Vivo

Building upon the excellent in vitro performance of TF‐Fe@LC, we first systematically evaluated its biosafety and biodistribution profiles before proceeding to antitumor assessment in tumor‐bearing mice. The initial in vitro safety assessment confirmed the exceptional biocompatibility of TF‐Fe@LC. First, cytotoxicity against normal human breast epithelial cells (MCF‐10A) was evaluated, showing minimal toxicity with cell viability >90% at 50 µg/mL (Figure S20). Additionally, excellent hemocompatibility was demonstrated at concentrations up to 1000 µg/mL, with hemolysis rates remaining below the 5% safety threshold (Figure S21A). Afterward, the in vivo biosafety of TF‐Fe@LC was evaluated. Balb/c mice were intravenously injected with TF‐Fe@LC at varying doses and monitored over 28 days. As shown in Figure S21B, while slight fluctuations in body weight occurred across groups during the observation period, all mice exhibited an overall upward trend, with no statistically significant differences between groups. All hematological parameters fell within normal physiological ranges, suggesting that TF‐Fe@LC caused no apparent adverse effects (Figure S21C–F). Notably, the blood urea nitrogen (BUN) level of mice in the 10 mg/kg dose group was significantly increased (Figure S21E), but still within the normal range (10.81–34.74 mg/dL). Interestingly, BUN levels in higher‐dose groups remained normal, implying that the elevation in the 10 mg/kg group might reflect individual variability rather than dose‐dependent toxicity. Histopathological analysis of major organs from TF‐Fe@LC‐treated mice revealed no observable tissue damage (Figure S22), further supporting the favorable biocompatibility of TF‐Fe@LC. Subsequently, the biodistribution and tumor‐targeting capability of TF‐Fe@LC were also verified in 4T1 tumor‐bearing mice. After intravenous injection of RhB‐labeled TF‐Fe@LC, major organs and tumor tissues were harvested at pre‐determined time intervals for fluorescence imaging. Fluorescent signals localized predominantly in the liver, spleen, and tumor sites within 4 h post‐injection, with tumor fluorescence intensity peaking at 24 h (Figure S23). Notably, persistent fluorescence retention was observed in the liver, spleen, and tumors even at 48 h post‐injection, demonstrating that TF‐Fe@LC achieves effective tumor accumulation while undergoing gradual systemic clearance. To quantify its systemic pharmacokinetics, the plasma elimination half‑life (t_1_/_2_) of TF‑Fe@LC was determined in healthy Sprague‑‐Dawley rats following a single intravenous dose. The mean elimination half‑life was calculated to be 6.5 h (Figure S24), indicating a favorable circulation time that supports its sustained tumor targeting.

**FIGURE 5:**
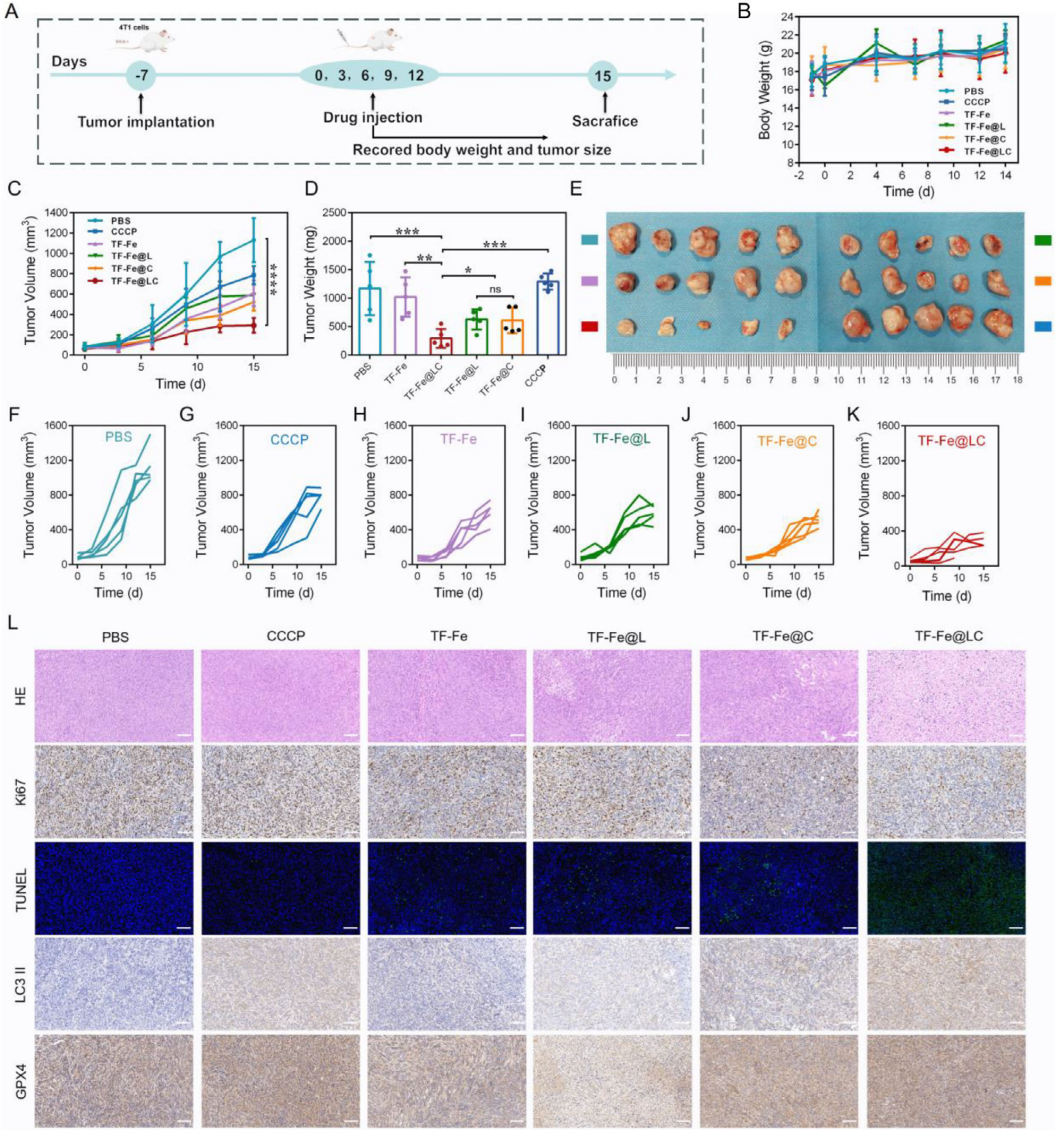
In vivo antitumor therapy of TF‐Fe@LC. (A) Schematic illustration of 4T1 tumor model establishment and treatment protocol. (B) Body weight of mice after various treatments during the therapeutic period. (C) Tumor volume changes of mice after various treatments during the therapeutic period. (D) Quantitative analysis of final tumor weights. (E) Representative excised tumors from each treatment group. The individual tumor growth curve of mice in (F) PBS, (G) CCCP, (H) TF‐Fe, (I) TF‐Fe@L, (J) TF‐Fe@C, (K) TF‐Fe@LC. (L) Representative images of tumor sections stained with H&E, TUNEL immunofluorescence, and IHC for Ki‐67 (proliferation marker), LC3‐II (autophagosome marker), and GPX4 (ferroptosis marker) following different treatment regimens. Scale bar: 100 µm. All measurements were performed in mice (*n* = 5 per group). ^*^p < 0.05, ^**^p < 0.01, ^***^p < 0.001, ^****^p < 0.0001.

Thereafter, the antitumor efficacy of TF‐Fe@LC was assessed on 4T1 tumor‐bearing mice following five intravenous administrations injected every three days (Figure 5A). The mice were randomly subdivided into six groups: (1) PBS (Control); (2) CCCP; (3) TF‐Fe; (4) TF‐Fe@L; (5) TF‐Fe@C; (6) TF‐Fe@LC. As shown in Figure 5B, no significant body weight variations were observed among the experimental groups during the treatment regimen. Tumor growth curves during the therapeutic period (Figure 5C,F–K) revealed rapid progression in both the PBS and CCCP groups. In contrast, the four other treatment groups demonstrated significant tumor suppression compared to the PBS group. Notably, the TF‐Fe@LC group displayed the most pronounced inhibition of tumor progression, as further validated by reduced mean tumor weight (Figure 5D) and representative tumor images (Figure 5E). Besides, histopathological analysis of major organs (heart, liver, spleen, lungs, and kidneys) and tumor tissues was performed. H&E staining results revealed no adverse effects on normal tissues in treated groups (Figure S25), while distinct cellular damage was observed in tumor tissues of the TF‐Fe@LC group (Figure 5L). TUNEL immunofluorescence staining and Ki67 immunohistochemistry were further carried out to confirm the antitumor effect of TF‐Fe@LC. Specifically, tumor tissues in the TF‐Fe@LC group exhibited the strongest green fluorescence signals (TUNEL), alongside a significant reduction in Ki67‐positive cells (Figure 5L), indicating robust inhibition of tumor cell proliferation. Moreover, the levels of autophagy and ferroptosis in tumor tissues were assessed by immunohistochemical staining. Immunohistochemistry (IHC) analysis revealed pronounced LC3 II overexpression in tumor tissues of the TF‐Fe@LC group (Figure 5L). This observation, together with autophagosome accumulation detected by Bio‐TEM (Figure S18) and sustained p62 protein level confirmed via Western blot (Figure 4E), demonstrated that TF‐Fe@LC simultaneously activated and blocked autophagy flux. Notably, IHC analysis demonstrated significant GPX4 downregulation in TF‐Fe and TF‐Fe@L groups versus the control group, whereas TF‐Fe@LC‐treated tumors retained GPX4 levels comparable to the control group. Collectively, the absence of ferroptosis‐related biomarkers (Figure 3H,I,L) suggested that ferroptosis made a minimal contribution to the antitumor efficacy of TF‐Fe@LC.

### 2.5 In Vivo Assessment of ICD Induction of TF‐Fe@LC and Its Combined Antitumor Efficacy with αPD‐L1

**FIGURE 6:**
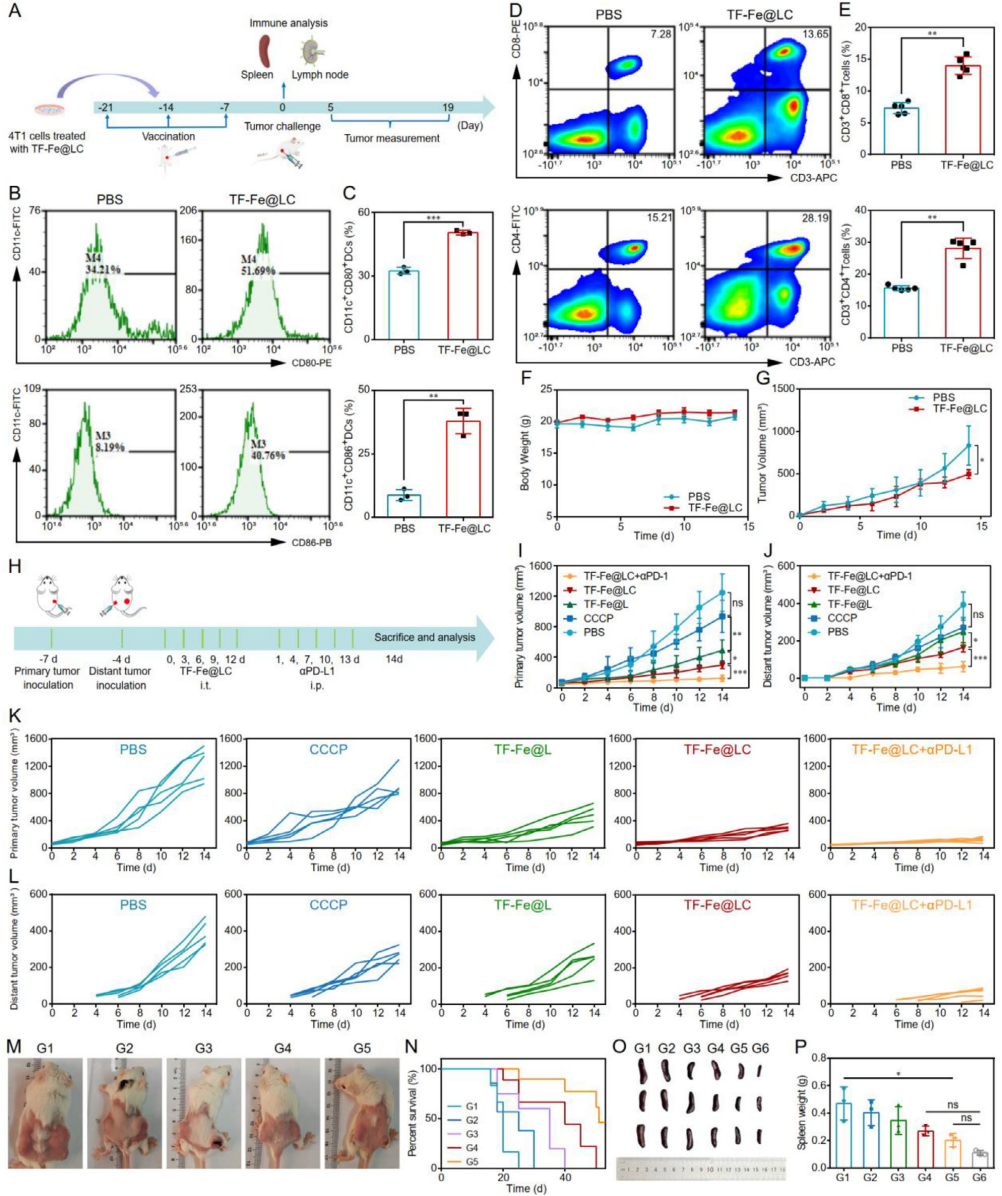
In vivo vaccination and bilateral tumor therapy. (A) Schematic illustration of in vivo tumor vaccination and rechallenge experimental design. Representative flow cytometric plots of (B) DC maturation markers (CD11c^+^CD80^+^/CD86^+^) in the lymph nodes, (D) CD4^+^ (CD3^+^CD4^+^) and CD8^+^ (CD3^+^CD8^+^) T cell activation in spleens post triple vaccination. (C) Quantification of DCs maturation in (B). (E) CD4^+^ (CD3^+^CD4^+^) and CD8^+^ (CD3^+^CD8^+^) T cell populations in (D). (F) Body weight changes in mice post‐tumor rechallenge. (G) Tumor growth curves following tumor rechallenge in vaccinated mice (*n* = 5). (H) Experimental schedule for combination therapy of TF‐Fe@LC with 𝛼PD‐L1 in bilateral 4T1 model. (I) Primary and (J) distant tumor growth curves across treatment groups (*n* = 5). (K,L) Individual tumor volume trajectories of primary (K) and distant (L) tumors in each mouse across treatment groups. (M) Representative photographs of bilateral tumor‐bearing mice after different treatments. (N) Post‐treatment survival rates in mice across different therapeutic groups (*n* = 6). (O) Photographs of the isolated spleen tissues and (P) spleen weight of the mice after different treatments (G1: PBS; G2: CCCP; G3: TF‐Fe@L; G4: TF‐Fe@LC; G5: TF‐Fe@LC+𝛼PD‐L1; G6: normal, *n* = 3). ^*^p < 0.05, ^**^p < 0.01, ^***^p < 0.001, ^****^p < 0.0001, ns: not significant.

Motivated by the prominent ICD induction in vitro and potent antitumor efficacy in vivo of TF‐Fe@LC, we sought to investigate whether ICD could be activated in vivo by TF‐Fe@LC and facilitate the abscopal effect through systemic immunity activation. In vivo tumor vaccination and rechallenge experiments were initially conducted to validate ICD [47]. In a nutshell, Balb/c mice received three weekly injections of TF‐Fe@LC‐treated 4T1 dead cells into the inguinal lymph node‐draining region. Half of the mice were sacrificed for flow cytometry analysis of spleen and lymph nodes, while the remaining half were challenged with fresh 4T1 cells on the contralateral dorsal flank to monitor tumor growth (Figure 6A). The mature dendritic cells (DCs) from draining lymph nodes near the injection site were analyzed at 7 days post‐final vaccination. Compared to the PBS group (unvaccinated), the TF‐Fe@LC‐treated 4T1 dead cells led to a higher level of DC maturation as the percentages of CD11c^+^CD80^+^ DCs and CD11c^+^CD86^+^ DCs increased to 51.7% and 40.8%, respectively (Figure 6B,C). It is well‐known that mature DCs prime T cell activation through antigen presentation and co‐stimulatory signals, thereby potentiating their antitumor immune response [48, 49]. As shown in Figure 6D,E, and Figure S26, the vaccination enabled obvious proliferation of splenic CD4^+^ (28.2%) and CD8^+^ T cells (13.7%). Additionally, PBS‐treated cells showed negligible suppression of rechallenged tumor growth, indicating poor tumor immunogenicity. Conversely, vaccinated mice with dying cells treated by TF‐Fe@LC showed delayed tumor progression (Figure 6G; Figure S27). Furthermore, no adverse effects on body weight were observed in vaccined mice during the observation period (Figure 6F). These results validated that TF‐Fe@LC‐induced paraptosis generated highly immunogenic dying cells, serving as potent tumor vaccines to stimulate systemic antitumor immunity. Overall, this unique mechanism simultaneously drove potent tumor elimination via Mito‐ER stress cascades and systemic immune activation through ICD promotion.

The robust ICD and consequent antitumor immunity elicited by TF‐Fe@LC highlight a strategic advantage of employing paraptosis as the death modality. Unlike apoptosis, a programmed cell death process that generally does not trigger inflammatory responses, paraptosis offers distinct mechanistic benefits for immunotherapy. First, paraptosis is intrinsically linked to severe endoplasmic reticulum (ER) and mitochondrial stress, as these are potent stressors that induce canonical ICD signals such as calreticulin exposure and HMGB1 release [50]. Thus, by triggering paraptosis, TF‐Fe@LC directly engages a cell death pathway whose upstream cause is a powerful driver of immunogenicity. Second, the proteotoxic and metabolic catastrophe characteristic of paraptosis leads to a more prolonged and disorganized cell death process compared to the rapid, caspase‐executed apoptosis. This may result in a sustained and amplified emission of DAMPs, creating a more potent and enduring immunogenic stimulus for dendritic cell activation and antigen presentation, as evidenced by our strong DC maturation and T‑cell priming data. Finally, paraptosis provides a crucial bypass to apoptosis resistance [51], a common escape mechanism in advanced cancers, thereby expanding the therapeutic reach of ICD‑based strategies. Therefore, the induction of paraptosis via multi‑organelle stress represents not merely an alternative way to kill tumor cells, but a deliberate strategy to unlock a particularly potent form of ICD that is deeply integrated with metabolic reprogramming and organelle dysfunction, offering a promising avenue for combination cancer immunotherapy.

Building on the above observations, we delved into an in vivo study combining TF‐Fe@LC‐induced paraptosis with PD‐L1 checkpoint blockade to interrogate systemic antitumor immunity and abscopal responses in a bilateral tumor model. The 4T1 tumor‐bearing mice were randomized into five experimental cohorts: PBS, CCCP, TF‐Fe, TF‐Fe@LC and TF‐Fe@LC+αPD‐L1. After the primary tumor volumes reached 60–80 mm^3^, the mice were treated as predefined protocol (Figure 6H). Daily monitoring of body weight in all groups of mice during the treatment period revealed negligible fluctuations (Figure S28). Following five treatment cycles, results depicted in Figure 6I–L revealed significant suppression of both primary and distant tumors in the TF‐Fe@LC group. Upon combination with αPD‐L1, the inhibition rates peaked at 90.2% (primary) and 84.5% (distant), respectively. Therapeutic efficacy was visually illustrated by the representative photographs of tumor‐bearing mice (Figure 6M) and the excised tumor tissues (Figure S29) post‐treatment. Encouraged by the promising therapeutic outcomes, the survival analysis was conducted to assess the long‐term efficacy of TF‐Fe@LC+αPD‐L1. As depicted in Figure 6N, mice treated with TF‐Fe@LC+αPD‐L1 demonstrated a prolonged survival time compared to the other groups. Notably, half of the mice in the TF‐Fe@LC+αPD‐L1 group survived beyond 50 days, further supporting the superior anti‐tumor efficacy and enhanced survival benefits. These findings collectively validated that such a combination of TF‐Fe@LC and αPD‐L1 could effectively elicit abscopal effects through the combined action of oxidative stress amplification and immune checkpoint reprogramming. This aligns with the established paradigm wherein ICD inducers can potentiate antitumor immune responses, as evidenced by recent studies combining ICD inducers with anti‐PD‐L1 antibodies [52]. As previously reported [53, 54], significant splenomegaly triggered by extramedullary hematopoiesis would be induced after tumor implantation. Treatment with TF‐Fe@LC+αPD‐L1 significantly reduced spleen size compared to enlarged spleens in other groups (Figure 6O). Although spleen weights did not fully return to normal levels, this combination therapy achieved a 56.8% reduction relative to the control group (Figure 6P), where mean spleen mass was 4.27‐fold higher than that in normal mice. This attenuation of splenomegaly implied alleviated immunosuppression, potentially enhancing antitumor immunity.

### 2.6 Enhanced Immune Activation of TF‐Fe@LC Combined With αPD‐L1

**FIGURE 7:**
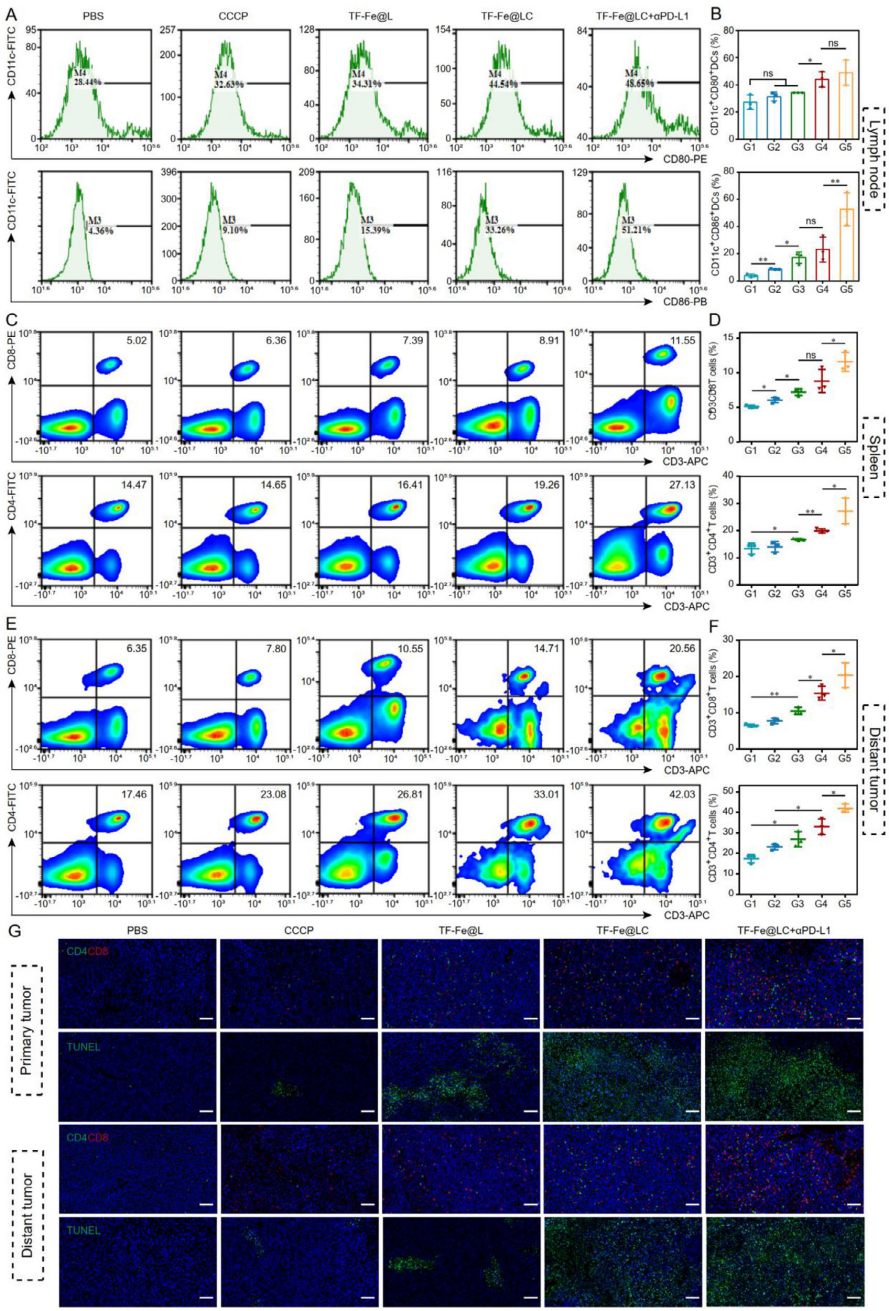
Immunological evaluation of mice following combined therapy with TF‐Fe@LC and αPD‐L1 antibody. (A) Representative flow cytometric plots of DC maturation markers (CD11c^+^CD80^+^/CD86^+^) in tumor‐draining lymph nodes after different treatments. (B) Quantification of DCs maturation in (A) (*n* = 3). (c) CD4^+^ (CD3^+^CD4^+^) and CD8^+^ (CD3^+^CD8^+^) T cell activation in spleens after different treatments. (D) CD4^+^ (CD3^+^CD4^+^) and CD8^+^ (CD3^+^CD8^+^) T cell populations in (C). (E) CD4^+^ (CD3^+^CD4^+^) and CD8^+^ (CD3^+^CD8^+^) T cell activation in distant tumors after different treatments. (f) CD4^+^ (CD3^+^CD4^+^) and CD8^+^ T cell (CD3^+^CD8^+^) populations in (E). (G) Immunostaining of TUNEL and CD4^+^and CD8^+^ cells in tumor slices from primary and distant sites. Scale bar, 100 µm, CD4 (green), CD8 (red), TUNEL (green), and nucleus (blue, DAPI). ^*^p < 0.05, ^**^p < 0.01, ns: not significant.

After identifying the favorable abscopal anti‐tumor effects of TF‐Fe@LC together with αPD‐L1, the immune activation after various treatments was further evaluated in vivo. To assess systemic immune responses, DCs from tumor‐draining lymph nodes (TdLNs), splenic immune cells, and tumor‐infiltrating lymphocytes in bilateral tumors were isolated and subsequently analyzed via flow cytometry. As shown in Figure 7A,B, the TF‐Fe@LC combined with αPD‐L1 treatment group exhibited a significantly higher percentage of mature DCs (CD80^+^CD86^+^) compared to other groups. Activated DCs exhibited enhanced antigen‐presenting capacity, triggering robust CD8^+^ cytotoxic T cell activation. This led to significant expansion of both CD4^+^ and CD8^+^ T cell populations in the spleen following combination therapy with TF‐Fe@LC and anti‐PD‐L1 (Figure 7C,D). Furthermore, combinatorial treatment induced the most pronounced intratumoral T cell infiltration, with CD4^+^/CD8^+^ lymphocyte populations in primary tumors (Figures S30 and S31) and distant tumors (Figure 7E,F). The CD4^+^ (green) and CD8^+^ (red) T lymphocyte infiltration in tumor tissues was also examined by immunofluorescence staining. The images revealed a sparse distribution of CD4^+^ and CD8^+^ T cells in PBS and CCCP groups. In contrast, TF‐Fe@LC alone and its combination with PD‐L1 blockade exhibited markedly enhanced lymphocyte infiltration, with the combinatorial regimen demonstrating prominent CD8^+^ T cell dominance (Figure 7G). These findings strongly support that coordinated TF‐Fe@LC and anti‐PD‐L1 therapy effectively potentiated cytotoxic T lymphocyte infiltration into tumor lesions. As reported in prior studies [55], the regulatory T cells (Tregs, CD4^+^CD25^+^Foxp3^+^), as a major barrier to anti‐cancer immunity, depend on lactate uptake to maintain their potent immunosuppressive capacity [56, 57]. Consistent with this mechanism, we first confirmed that TF‐Fe@LC treatment significantly reduced the intratumoral lactate concentration (Figure S32), thereby impairing the induction of Tregs and undermining their immunosuppressive function. Notably, the combination of TF‐Fe@LC and PD‐L1 blockade further induced a significant decline of Tregs in distant tumors (Figure S33). These findings suggest that, beyond lactate consumption, the stronger suppression of Tregs by TF‑Fe@LC likely stems from its superimposed oxidative stress attack [58]. Together, they combine with PD‐L1 blockade to relieve tumor immunosuppression. Furthermore, the levels of inflammatory cytokines were also assessed in tumor tissues. The combination of TF‑Fe@LC with αPD‑L1 markedly upregulated the production of IFN‑γ and TNF‑α (Figure S34), indicating that paraptosis induced by TF‑Fe@LC, together with PD‑L1 blockade, further promotes the release of DAMPs and related inflammatory mediators. Ultimately, the tumor suppression of TF‐Fe@LC combined with αPD‐L1 was further investigated by TUNEL staining to observe histological changes. Consistent with the results of the tumor inhibition effect, a strikingly enhanced green fluorescence was observed in the combined treatment group (Figure 7G), with faint signals observed in the PBS group, indicating extensive cellular death within the tumor. Collectively, these results indicated that the induction of the paraptosis pathway by TF‐Fe@LC could activate the ICD, thereby significantly augmenting the host immune response. In combination with αPD‐L1, this combination achieved robust suppression of distant tumor progression via enhanced CD8^+^ T cell infiltration and Treg downregulation.

## 3 Conclusion

In summary, the engineered iron‐catalyzed nanoplatform (TF‐Fe@LC) activated paraptosis through multi‐organelle stress amplification, initiating with oxidative outburst, propagating via autophagic flux blockade, and culminating in organelle damage to establish a non‐apoptotic demise pathway. The acidic TME triggered the dissociation of Fe^3^^+^‐TA coordination networks, enabling targeted release of LOD, CCCP, and Fe ions. The LOD‐mediated enzymatic conversion of lactate to H_2_O_2_ not only alleviated the immunosuppressive TME, but also provided a sustainable substrate pool for Fe^3^^+^/Fe^2^^+^‐catalyzed Fenton reactions, generating cytotoxic hydroxyl radicals (·OH) to initiate oxidative cascades. Crucially, CCCP synergized with CDT to destabilize mitochondrial membrane potential, exacerbating ROS overproduction and propagating oxidative damage to catastrophic levels. The in vitro assays revealed that TF‐Fe@LC treatment dramatically increased intracellular ROS levels. Leveraging this oxidative escalation, cell viability assays demonstrated significant tumor cell viability reduction primarily through ROS‐mediated non‐canonical cell death pathways. Severe mitochondrial dysfunction was observed in the TF‐Fe@LC‐treated group with the decrease of mitochondrial membrane potential and ATP exhaustion. Furthermore, ROS overproduction activated the PERK‐p‐eIF2α‐ATF4 axis of ER stress, while concurrently inhibiting autophagosome‐lysosome fusion to block autophagic flux. This dual perturbation exacerbated intracellular oxidative stress, culminating in caspase‐independent paraptosis characterized by cytoplasmic vacuolization and ER/mitochondrial collapse. More importantly, TF‐Fe@LC‐induced paraptosis elicited robust ICD by contributing to releasing large amounts of DAMPs to recruit DCs for activating antitumor immunity. The combination with αPD‐L1 antibodies stimulated stronger systemic immunity, and suppressed distant tumor progression by supporting T cell infiltration and Tregs downregulation in distant tumors. This study elucidates that porous iron‐based nanocatalysts provoke organelle‐damaging paraptosis through ROS cascade amplification, with combined checkpoint inhibitors significantly potentiating tumor eradication rates. These findings establish a new paradigm for programming cell‐death modalities in nanomedicine and pioneer therapeutic avenues against apoptosis‐resistant malignancies.

## 4 Experimental Section

### 4.1 Materials

Ferric chloride hexahydrate (FeCl_3_⋅6H_2_O), urea (CO(NH_2_)_2_), citric acid (C_6_H_8_O_7_), sodium polyacrylate (PAAS), carbonyl cyanide m‐chlorophenylhydrazone (CCCP), and lactate oxidase (LOD) were purchased from Sigma–Aldrich (Germany). Ethylene glycol, 3,3’,5,5’‐tetramethylbenzidine (TMB), and tannic acid (TA) were purchased from Sinopharm Chemical Reagent Co. Ltd. (China). BCA protein assay kits, hydrogen peroxide assay kits, 2',7'‐dichlorofluorescein diacetate (DCFH‐DA), JC‐1 (5,50, 6,60‐tetrachloro‐1, 10,3,30‐tetraethylbenzimidazolecarbocyanine iodide), Enhanced ATP Assay Kit, Lactate Assay Kit with WST‐8, Calcein AM/PI Kit, ER‐Tracker Green, MitoTracker Red CMXRos, lipid peroxidation (MDA) assay kits, Hanks' balanced salt solution (HBSS), and Hoechst 33342 were purchased from Beyotime (China). Ferrostatin‐1, Z‐VAD‐FMK, necrosulfonamide (NSA), N‐acetylcysteine (NAC), cycloheximide (CHX), necrostatin‐1 (Ner), and αPD‐L1 were purchased from Selleck (China). Chloroquine (CQ), 3‐methyladenine (3‐MA), Hydrogen Peroxide Content Assay Kit, and paraformaldehyde were obtained from Solarbio Technology Co., Ltd. (Beijing, China). Mammary Epithelial Cell Growth Medium BulletKit (MEGM) was obtained from Lonza Bioscience (Switzerland). Antibodies against calreticulin (CRT), high‐mobility group box 1 (HMGB1), LC3 I/II, and p62/SQSTM1 were acquired from Abcam (Shanghai, China). Antibodies against BiP, IRE1α, PERK, phospho‐PERK (p‐PERK), ATF4, eIF2α, and phospho‐eIF2α (p‐eIF2α) were purchased from Cell Signaling Technology (USA). Non‐permeabilizing red blood cell lysis buffer was obtained from Beckman Coulter (USA). The tumor tissue dissociation kit was purchased from Miltenyi Biotec (Germany). Antibodies against CD11c‐FITC, CD80‐PE, CD86‐PB, CD3‐APC, CD4‐FITC, CD8‐PE, CD25‐PE, and Foxp3‐APC were purchased from Biolegend (USA).

### 4.2 Cell Culture and Cell Viability Assay

The 4T1 murine breast cancer cell line and the human normal breast epithelial cell line MCF‐10A were purchased from the cell bank of the Chinese Academy of Sciences, Shanghai. Both cell lines were maintained at 37°C in a humidified 5% CO_2_ incubator, with 4T1 cultured in RPMI‑1640 containing 10% FBS and 1% penicillin/streptomycin, and MCF‑10A cultured in MEGM supplemented with the provided BulletKit. For CCK‑8 assays, cells were seeded in 96‑well plates at a density of 1 × 10^5^ cells per well overnight, and treatments were applied in fresh medium. 4T1 cells were treated with a range of concentrations of CCCP (0, 0.5, 1, 5, 10, 20 µg/mL), H_2_O_2_ (0, 12.5, 25, 50, 100, 200, 400, 800 µm), TF‑Fe (0, 25, 50, 100, 200, 400 µg/mL), TF‑Fe@L (50 µg/mL) and TF‑Fe@LC (50 µg/mL), with or without 12 mm lactate. MCF‑10A cells were treated with 5 µg/mL CCCP, 50 µg/mL TF‑Fe, TF‑Fe@L, and TF‑Fe@LC. After 24 h, cell viability was assessed using the CCK‑8 kit. For visualization of live/dead cells, treated 4T1 cells were stained with a Calcein‐AM/PI mixture and imaged by confocal laser scanning microscope (CLSM).

### 4.3 Establish of 4T1‐Bearing Mouse Model

Female Balb/c mice (5 weeks) were purchased from the Laboratory Animal Center, Air Force Medical University (Xi'an, China). All the animal experiments were conducted in accordance with the Guide for the Care and Use of Laboratory Animals and approved by the Institutional Animal Care and Use Committee (IACUC) of Air Force Medical University (No. KY251260). The in vivo tumor model was established by subcutaneous injection of 4T1 cell into the female Balb/c mice (6 weeks, 20 ± 2 g). The tumor volume was calculated using the formula: length × width^2^/2.

### 4.4 Statistical Analysis

All experimental data were shown as mean ± standard deviation (SD). Data were analyzed for statistical significance using parametric tests: Student's *t*‐test (for two‐group comparisons) or one‐way ANOVA (for multi‐group comparisons), indicated by ^*^p < 0.05, ^**^p < 0.01, and ^***^p < 0.001.

## Funding

The National Natural Science Foundation of China (32171388), Shaanxi Provincial Key Research and Development Program (Key Industry Innovation Chain Project, 2023‐ZDLSF‐57, 2022‐ZDLSF05‐17; General Project, 2023‐YBSF‐504), the Natural Science Foundation of Shaanxi Province (2024JC‐YBQN‐0941), the “Clinical Medicine + Pharmacy” Research Project of Xijing Hospital (LHJJ2023‐YX12), Xijing Hospital Interdisciplinary Medical Research Promotion Special Program (XJZT24JC05).

## Conflicts of Interest

The authors declare no conflicts of interest.

## Supporting information




**Supporting File**: advs74593‐sup‐0001‐SuppMat.doc.

## Data Availability

The data that support the findings of this study are available from the corresponding author upon reasonable request.
