# Supplementary material for: Multi‐Organelle Stress‐Induced Paraptosis by a ROS‐Amplifying Nanocatalyst for Enhanced Cancer Immunotherapy
Source: Adv Sci (Weinh). 2026 Mar 5;13(26):e20031. doi: 10.1002/advs.202520031 (PMC13159107; doi:10.1002/advs.202520031)
Supplement: Supplementary file 1 — Supporting File: advs74593‐sup‐0001‐SuppMat.doc. [file ADVS-13-e20031-s001.doc]

**Supporting Information**

**Multi-Organelle Stress-Induced Paraptosis by a ROS-Amplifying Nanocatalyst for Enhanced Cancer Immunotherapy**

Zhe Yu^†^, Haozhe Ren^†^, Hua Li^†^, Jinghan Zhao, Miaomiao Cao, Tian Tang, Youbei Qiao, Liting Chen, Tiehong Yang, and Hong Wu*

^†^These authors contributed equally to this work.

Z. Yu, J. Zhao, M. Cao, T. Tang, Y. Qiao, T. Yang, H. Wu

Department of Pharmaceutical Analysis, School of Pharmacy, the Fourth Military Medical University, Xi’an 710032, China

E-mail: [wuhong@fmmu.edu.cn](mailto:wuhong@fmmu.edu.cn) (H. W)

H. Ren

School of Stomatology, Lanzhou University, Lanzhou 730000, China

H. Li

Department of Chinese Materia Medica and Natural Medicines, School of Pharmacy, the Fourth Military Medical University, Xi’an 710032, China

L. Chen

Department of Radiation Oncology, Xijing Hospital, Air Force Medical University, the Fourth Military Medical University, Xi’an 710032, China

**Keywords:** Chemodynamic therapy; Paraptosis; Mitochondrial dysfunction; Endoplasmic reticulum stress; Autophagy; Immunogenic cell death

**Experimental Section**

***Synthesis of ultrasmall nanocrystal-assembled porous Fe_3_O_4_ nanoclusters (P-Fe NCs)***

The monodisperse superparamagnetic Fe_3_O_4_ NCs were prepared through a modified solvothermal method reported previously ^[1,2]^. In brief, FeCl_3_∙6H_2_O (0.27 g), sodium citrate (1.2 g) and urea (0.36 g) were dissolved in distilled water (60 mL) with continuous stirring under anaerobic conditions. Then, PAAS (0.3 g) was added under vigorous stirring until it was totally dissolved. The solution was transferred to a Teflon-lined autoclave and maintained at 200 °C for 4 h. The product was collected by magnetic separation, washed with distilled water for three times.

Next, the porous structure was adjusted by using the citric acid (CA) etching method. The black magnetite precipitate was dispersed in 25 ml of ethylene glycol using an ultrasonic bath, to which 0.5 M citric acid was added at 160 °C by an oil bath, protected by nitrogen gas throughout the experiment and mechanically stirred at 400 r/min. After etching a certain time, the precipitate was isolated and repeatedly washed with ultrapure water until the monodisperse Fe_3_O_4_ nanoclusters were obtained. The nanoclusters were designated as P-Fe NCs and stored for further application.

***Synthsis of Fe^3+^-TA-P-Fe@LOD/CCCP (TF-Fe@LC)***

P-Fe solution (500 μg/mL), LOD solution (100 U), and CCCP solution (100 μg/mL) were mixed. Then, 20 µL of FeCl_3_∙6H_2_O solution (24 mM) and 20 µL TA solution (24 mM) were sequentially added in the above solution with continuous vortex. After that, the TF-Fe@LC was washed with ultrapure water and collected by centrifugation. The TF-Fe@L and TF-Fe@C were fabricated similarly under the absence of CCCP and LOD, respectively. After each wash, the supernatants were collected to quantify the eluted LOD or CCCP concentration by BCA protein assay kit or UV-vis spectrometry. The drug loading rates were calculated by the differential subtraction method.

***Synthsis of RhB conjugated P-Fe_3_O_4_***

To prepare the RhB-labled P-Fe_3_O_4_, the P-Fe_3_O_4_ solution was added into a 20 mL mixture solution of EDC (2 mg) and NHS (2 mg). After stirring for 4 h, 20 mL of Rhodamine-NH_2_ (0.5 mg/mL) solution was added into the mixture. After reacting for 48 h, the mixture solution was repetitively washed and centrifuged to remove the unreacted RhB, EDC, and NHS to obtain RhB-labled P-Fe_3_O_4_.

***In vitro biodegradability of*** ***P-Fe NCs***

P-Fe NCs (10 mg) was dissolved in 40 mL of PBS buffer solutions (pH 5.0), agitated in a shaker at 37 °C, sampled as well as centrifuged at specific time intervals for TEM characterization.

***Fe^2+^ release of P-Fe NCs***

P-Fe NCs (1 mg/mL, 1 mL) were placed in PBS buffer solutions at different pH values (7.4, 5.5 and 4.5), agitated in a shaker at 37 °C. At specific time intervals, the samples were centrifuged and the supernatants were added with o-phenanthroline solution (10 mM). The absorbance at 510 nm of the supernatants were then measured by UV-vis spectrometry.

***Drug release detection*** ***in TF-Fe@LC***

Drug release profiles of CCCP and LOD from TF‑Fe@LC were systematically evaluated. For comparison, the release of CCCP from Fe@LC was also assessed. Briefly, Fe@LC and TF-Fe@LC were dispersed in PBS with different pH values (6.5, 5.0). At specific time intervals, the samples were taken out and washed by magnetic separation. The supernatants were collected for analysis. The concentration of CCCP was determined by measuring the absorbance at 370 nm and calculated using a standard curve. LOD concentration was quantified via a BCA protein assay kit. The cumulative release percentage was calculated relative to the total loaded amount.

***‧OH generation assay***

To investigate the generation of ‧OH, TMB was employed as the indicator. Briefly, P-Fe (25 μg/mL) and TMB (0.5 mM) were added upon the addition of H_2_O_2_ or lactic acid for 5 min at room temperature (RT). The absorbance changes at 650 nm were measured by UV-vis spectroscopy. The dependence of enzymatic activity under different pH values (pH 7.4, 6.5, 5.0, 4.5, and 3.6) and the enzymatic activity of different nanosystems (P-Fe, TF-Fe and TF-Fe@LC) were studied under the same conditions. The Michaelis–Menten kinetic curves of the P-Fe NCs were acquired by plotting the initial velocities against the concentrations of H_2_O_2_. The maximal velocity (V_max_) and Michaelis–Menten constant (K_m_) were calculated by creating Lineweaver–Burk plots. Furthermore, electron paramagnetic resonance (ESR) spectroscopy with 5, 5-dimethyl-1-pyrroline N-oxide (DMPO) as a spin trap was also used to capture ⋅OH.

***Hemolysis test***

Blood was taken from healthy rabbits and serum was removed by centrifugation three times. Increasing concentrations of TF-Fe@LC (0, 25, 50, 100, 200, 500, 1000 μg/mL) were added into the above suspension and incubated for 1 h at 37 °C. Then, the supernatants from these samples were collected by centrifugation and measured at the absorbance of 541 nm using a UV-vis spectrophotometer.

***Intracellular endocytosis analysis***

For CLSM observation, 4T1 cells were seeded in 35 mm confocal dishes overnight. Then the culture media were replaced by RhB-labled NPs (50 μg/mL). After different co-incubation times (1, 2, 4 and 8 h), the cells were labeled with DAPI for 5 min in the dark. At the end of staining, cells were rinsed by PBS twice and inspected with the CLSM.

***Intracellular observation of ROS production***

4T1 cells were seeded in 35 mm confocal dishes overnight and then treated for 12 h with CCCP, TF-Fe, TF-Fe@L, or TF-Fe@LC (each at 50 µg/mL). Then, the cells were incubated with DCFH-DA for 30 min and observed by CLSM. For quantitative analysis, the same treatment and staining procedures were followed, after which the cells were harvested and analyzed by flow cytometry.

***Intracellular Measurement of Lactate Content***

Given that intracellular lactate concentration is a critical parameter for initiating the cascade reaction, we measured lactate levels to evaluate the catalytic function of TF-Fe@LC. 4T1 cells were seeded in 6-well plates overnight and then exposed to CCCP, TF-Fe, TF-Fe@L, or TF-Fe@LC (50 µg/mL) for the indicated duration. After treatment, cells were lysed, centrifuged, and the supernatant was collected for lactate quantification using a Lactate Assay Kit.

***Detection of Intracellular ATP level and Mitochondrial Transmembrane Potential***

The ATP levels of TF-Fe@LC treated 4T1 cells were tested by ATP assay kit. 4T1 cells were inoculated into 6-well plates and incubated overnight. After that, the culture media were treated with CCCP, TF-Fe, TF-Fe@L and TF-Fe@LC (50 µg/mL). The culture medium was aspirated and cells were blown to fully lysed. ATP content was measured by the enhanced ATP Assay Kit following the instructions and detected by a chemiluminescent analyzer.

Mitochondrial membrane permeability was determined using the fluorescent probe JC-1 dye. 4T1 cells were plated in 35 mm confocal dishes and treated with the aforementioned method. Then, 1 mL of JC-1 was added to the cells and incubated for 30 minutes, followed by CLSM observation.

***Study of the*** ***TF-Fe@LC-induced cell death pathways in 4T1 cells***

To elucidate the mechnism of TF-Fe@LC-induced cell death *in vitro*, 4T1 cells were preincubated in 96-well plates at the destiny of 10^5^ cells per well overnight. Cells were treated with TF-Fe@LC or with a combination of Ferrostatin-1 (2 μM), Z-VAD (10 μM), 3-MA (40 μM), necrosulfonamide (2 μM), necrostatin-1 (2 μM) for 24 h, and a CCK-8 assay was conducted to investigate the reversal effects of these inhibitors on TF-Fe@LC-induced 4T1 cell death.

***Determination of intracellular MDA content***

4T1 cells were inoculated into 6-well plates and incubated overnight. After that, the culture media were treated with CCCP, TF-Fe, TF-Fe@L and TF-Fe@LC (50 µg/mL). After 12 h, the culture medium was discarded and the cells were lysed according to the protocol of the MDA Assay Kit. The intracellular MDA content was measured at 532 nm using a microplate reader.

***Mechinsm of TF-Fe@LC-induced cell death***

A detailed assessment of the therapeutic mechanisms induced by TF-Fe@LC was conducted using transcriptomic analysis, Bio-TEM, organelle staining, and western blot assay. For transcriptomic analysis, total RNA was extracted using TRIzol reagent (Invitrogen) followed by DNase I treatment and purification with RNeasy Mini Kit (Qiagen). RNA sequencing was performed on an Illumina NovaSeq X platform. For Bio-TEM, 4T1 cells were treated with TF-Fe@LC for 0、4、8、12 h. At the pre-determined time, the cells were collected and fixed with 2.5% glutaraldehyde, followed by staining and sectioning for TEM observation. Then, 4T1 cells were treated with TF-Fe@LC for 12 h. The MitoTracker-Red or ER-Tracker Green was co-incubated with the cells for another 15 min to stain mitochondria or the endoplasmic reticulum, respectively. To perform western blot analysis, 4T1 cells were lysed with RIPA lysis buffer on ice bath and the corresponding proteins were accurately determined by BCA kit. Subsequently, the proteins were separated by 8-12% SDS-PAGE and transferred to poly (vinylidene fluoride) (PVDF) membrane for blocking. Thereafter, trimmed membranes were incubated overnight at 4 °C with various primary antibodies to LC3 I/II, p62, β-actin, PERK, p-PERK, eIF2α, p-eIF2α, ATF4, IRE1α and BiP at the recommended dilution ratios. After being washed with TBST solution, the membranes were hatched with the corresponding secondary antibody for 1 h. Finally, ECL detection reagent was employed for protein visualization, followed by Image J software for quantitative analysis.

***The investigation of immunogenic cell death* *in vitro***

4T1 cells were planted in confocal dishes and treated as mentioned above. After washing, the cells were fixed with 4% glutaraldehyde for 15 min at RT, subsequently permeabilized with 0.1% Triton X-100 for 10 min and blocking buffer (3% BSA) for 2 h at RT. Then, cells were hatched with indicated primary antibodies (CRT and HMGB1) and secondary antibody (ABflo® 488-conjugated Goat anti-Rabbit IgG). After washing with PBS, the cells were incubated with DAPI for immunofluorescence imaging.

***Assessment of dendritic cell maturation in vitro***

To evaluate the immunostimulatory capacity of TF-Fe@LC-induced dying cells, an *in vitro* DC maturation assay was performed. Conditioned medium (CM) was collected from 4T1 cells after a 12 h treatment with TF-Fe@LC (50 µg/mL) or control, followed by centrifugation and sterile filtration. DCs were then incubated with this CM (50%, v/v) for 24 h. Subsequently, DCs were harvested, stained with fluorescent antibodies against mouse CD11c, CD80, and CD86, and analyzed by flow cytometry. The percentage of CD11c^+^ cells co-expressing CD80 and/or CD86 was quantified to determine DC maturation.

***In vivo* *biodistribution and biosafety study***

When the tumor volume reached around 100 mm^3^, the mice were injected with RhB-labled P-Fe_3_O_4_ (12.5 mg/kg) intravenously. At the predetermined time (0, 4, 8, 24, 48 h), the mice were euthanized and the tumors and major organs were harvested for *ex vivo* fluorescence imaging.

For biosafety study, the healthy Balb/c mice were intravenously injected with PBS or TF-Fe@L/C (10、20、40 mg/kg). The body weight of each mouse was recorded every 2 days during the tested period. Blood was collected for blood routine and biochemistry analysis at day 28 after the injection. The levels of aspartate aminotransferase (AST), serum albumin (ALB), alkaline phosphatase (ALP), alanine aminotransferase (ALT), blood urea nitrogen (BUN) and creatinine were determined according to the standard protocols. Then, the major organs (heart, liver, spleen, lung, kidney) were performed for hematoxylin and eosin (H&E) staining.

***In vivo* *pharmacokinetic* *study***

RhB‑labeled TF-Fe@LC was intravenously administered to male Sprague‑Dawley rats. Approximately 100 μL of blood was collected from the orbital venous plexus at 0, 5 min, 10 min, 30 min, 1 h, 2 h, 4 h, 6 h, 8 h, 12 h, and 24 h post‑administration. The concentration of RhB labeled nanoplatform in plasma was measured using a fluorescence spectrophotometer. The plasma concentration‑time data were subsequently analyzed by non‑compartmental fitting with DAS 3.0 software, and the elimination half‑life (t₁/₂) was calculated from the fitted concentration‑time curve.

***In vivo* *antitumor study***

When the tumor volume reached around 80 mm^3^, the 4T1 cancer-bearing mice were randomly divided into indicated groups (n=5): PBS, CCCP, TF-Fe, TF-Fe@L, TF-Fe@C, and TF-Fe@LC (12.5 mg/kg). The drugs were intravenously (i.v.) administration every three days, during which both the tumor volume and the body weights were systematically recorded. On day 14, the major organs and tumors were collected for H&E, TUNEL, and Ki-67 staining to trace tumor cell death and proliferation levels. Furthermore, anti-GPX4 and anti-LC3B antibodies were used to assess the ferroptosis and autophagy levels in tumor tissues.

***In vivo* *tumor vaccination and rechallenge study***

Female Balb/c mice (5 week) were randomized into 2 groups (n=10 per group) with PBS as control and TF-Fe@LC as vaccinated group. Tumor vaccines were prepared by treating 4T1 cells with TF-Fe@LC for 24 h, washing twice with PBS, and collecting floating dying cells. PBS-treated live 4T1 cells served as non-immunogenic controls. The healthy mice were received three subcutaneous immunizations near the groin with corresponding tumor vaccines (1×10⁶ dying cells or PBS) on day -21, -14 and day -7. On day 0, half of the mice in each group were euthanized and their lymph nodes as well as spleen were extracted under sterile conditions and stored in ice PBS. The remaining mice in each group were rechallenged by right-flank inoculation with 1×10⁶ fresh 4T1 cells. Tumor volumes and body weight in rechallenged mice were monitored every other day.

***Antitumor Abscopal Effect***

A bilateral 4T1 subcutaneous tumor model was established in female Balb/c mice as follows: 1 × 10^6^ 4T1 cells were inoculated subcutaneously into the right hind flank to construct the primary tumors. After 3 days, 5.0 × 10⁵ 4T1 cells were implanted in the left hind flank to construct the distant tumors. When the primary tumor volume reached 50-80 mm³, the mice were randomly allocated into five groups (n=12): PBS, TF-Fe@L, TF-Fe@LC (12.5 mg/kg), and TF-Fe@LC + αPD-L1. The drugs were administered intratumorally to primary tumors, followed by intraperitoneal delivery of αPD-L1 (100 µg per mouse) 24 hours later. The treatment regimen comprised five administrations at 3-day intervals. Tumor volumes and body weights of mice were recorded every other day up to 14 days. At the end of treatment, a randomly selected subset of mice per group (n=6) was euthanized. Tumors and spleens were immediately excised, weighed, and photographed. Excised tumors underwent immunofluorescence staining (TUNEL assay) and immunohistochemical analysis to evaluate the cytotoxic T-cell infiltration in bilateral tumors. For survival analysis, the remaining mice were monitored for 55 days with Kaplan-Meier methodology.

***Intratumoral lactate concentration measurement***

When distant tumors in 4T1‑bearing mice reached 80–100 mm³, the mice were intravenously injected (12.5 mg/kg). 24 hours post‑injection, tumors were dissected, weighed, and homogenized. The homogenates were centrifuged, and the resulting supernatant was collected for lactate concentration measurement.

***Immune response in bilateral tumor model***

In addition to the aforementioned excised tumors and spleens, tumor-draining lymph nodes (TDLNs) were harvested and processed into single-cell suspensions. The obtained single-cell suspensions were further stained with different antibodies against the immune cells and analyzed by flow cytometry. T cells infiltration in the tumor and spleen were stained by anti-CD3-APC, anti-CD4-FITC and anti-CD8a-PE. DCs maturation in lymph nodes were stained by anti-CD11c-FITC, anti-CD80-PE, and anti-CD86-PB. Treg cells in distant tumor were sequentially stained for surface markers (anti-CD4-FITC and anti-CD25-PE) and intracellular marker (anti-Foxp3-APC) following fixation/permeabilization. For cytokines detection, the concentrations of TNF-α, and IFN-γ in serum were quantified by using ELISA kits.

***Statistical Analysis***

All experimental data were shown as mean ± standard deviation (SD). Data were analyzed for statistical significance using parametric tests: Student's t-test (for two-group comparisons) or one-way ANOVA (for multi-group comparisons), indicated by *^*^p* < 0.05, *^**^p* < 0.01 and *^***^p* < 0.001.

**Supporting Figures**


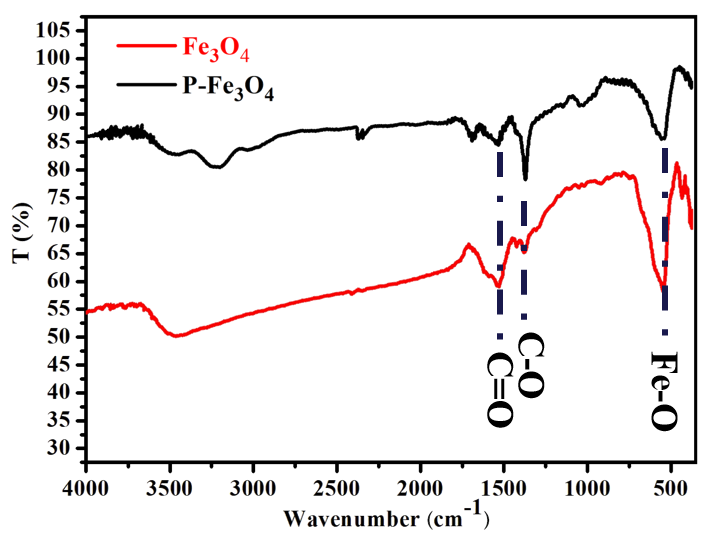


**Figure S1.** FT-IR of Fe_3_O_4_ and P-Fe NCs.

**
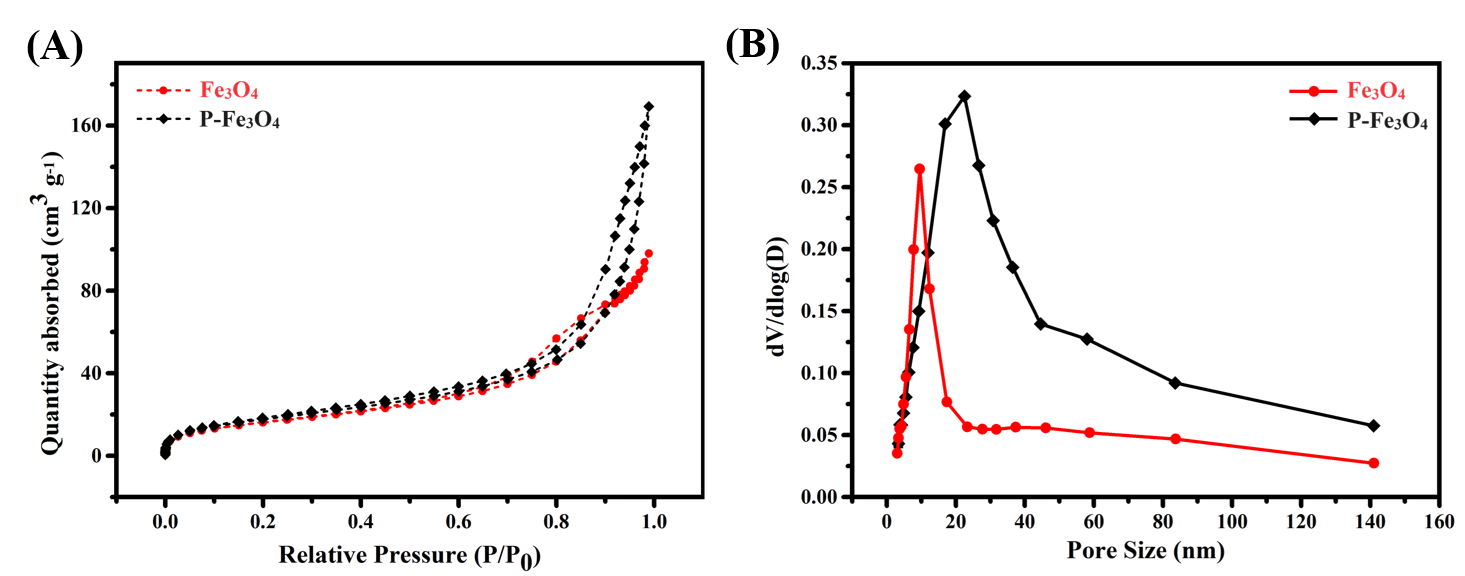
**

**Figure S2.** (A) N_2_ adsorption-desorption isotherms and (B) pore size distributions of Fe_3_O_4_ and P-Fe NCs.

| **Table S1.** BET surface area parameters | | | |
| --- | --- | --- | --- |
| Samples | BET surface area (m^2^/g) | BET pore volume (cm^3^/g) | BJH pore diameter (nm) |
| Fe_3_O_4_ | 59.56 | 0.148 | 9.585 |
| P-Fe | 98.47 | 0.258 | 16.892 |

**
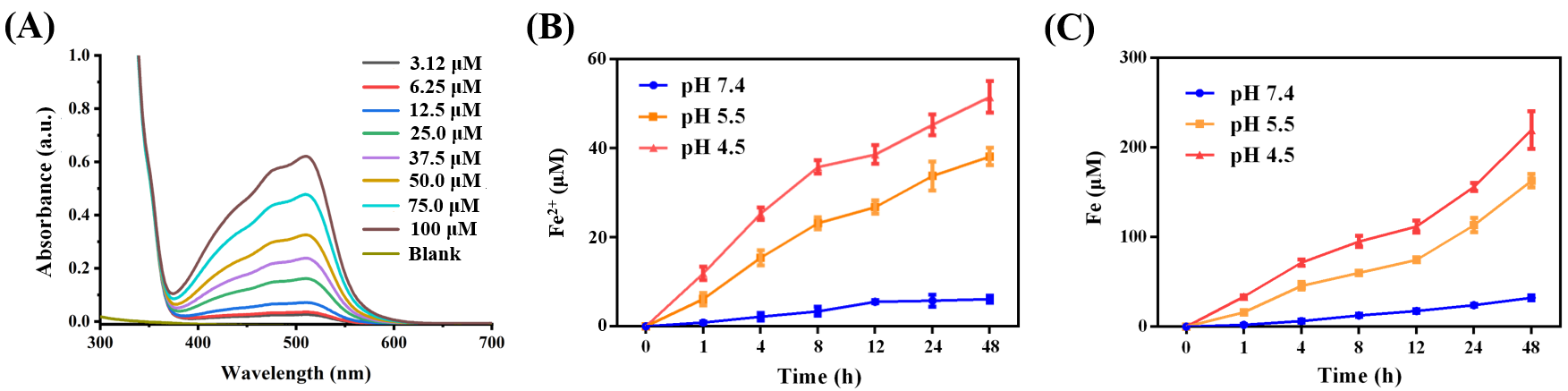
**

**Figure S3.** Iron release behavior. (A) UV-Vis spectra of Fe²⁺-phenanthroline complexes at varying iron concentrations. (B) Release kinetics of Fe^2+^ from P-Fe NCs at varying pH (n=3). (C) The cumulative release profile of total iron ions from P-Fe NCs under varying pH conditions (n=3).

**
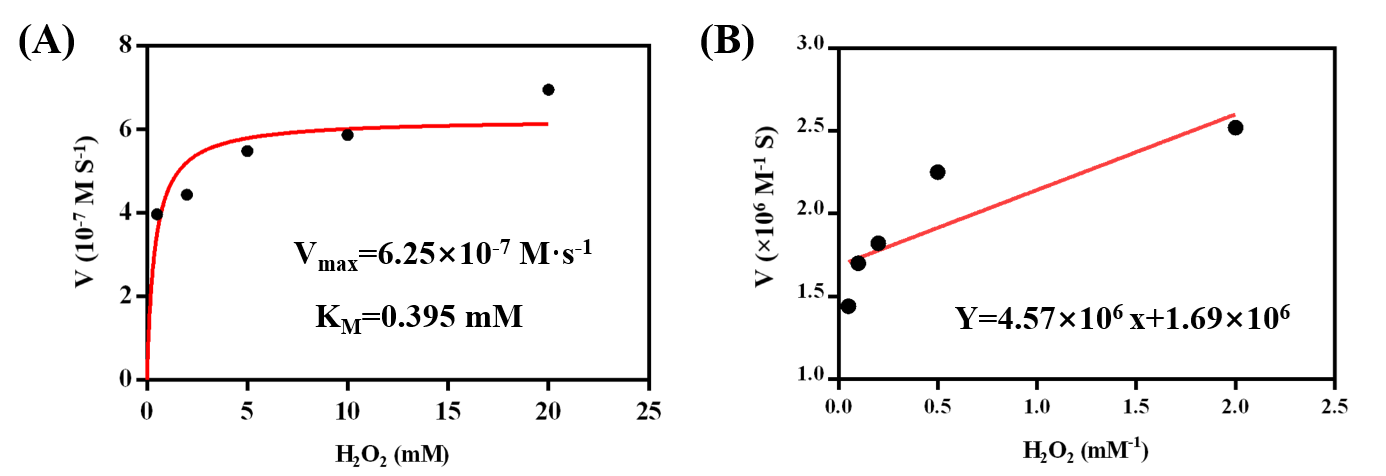
**

**Figure S4.** Catalytic performances of P-Fe NCs using H₂O₂ as substrate *in vitro*. (A) Michaelis-Menten kinetics and (B) Lineweaver-Burk plotting of the P-Fe NCs.


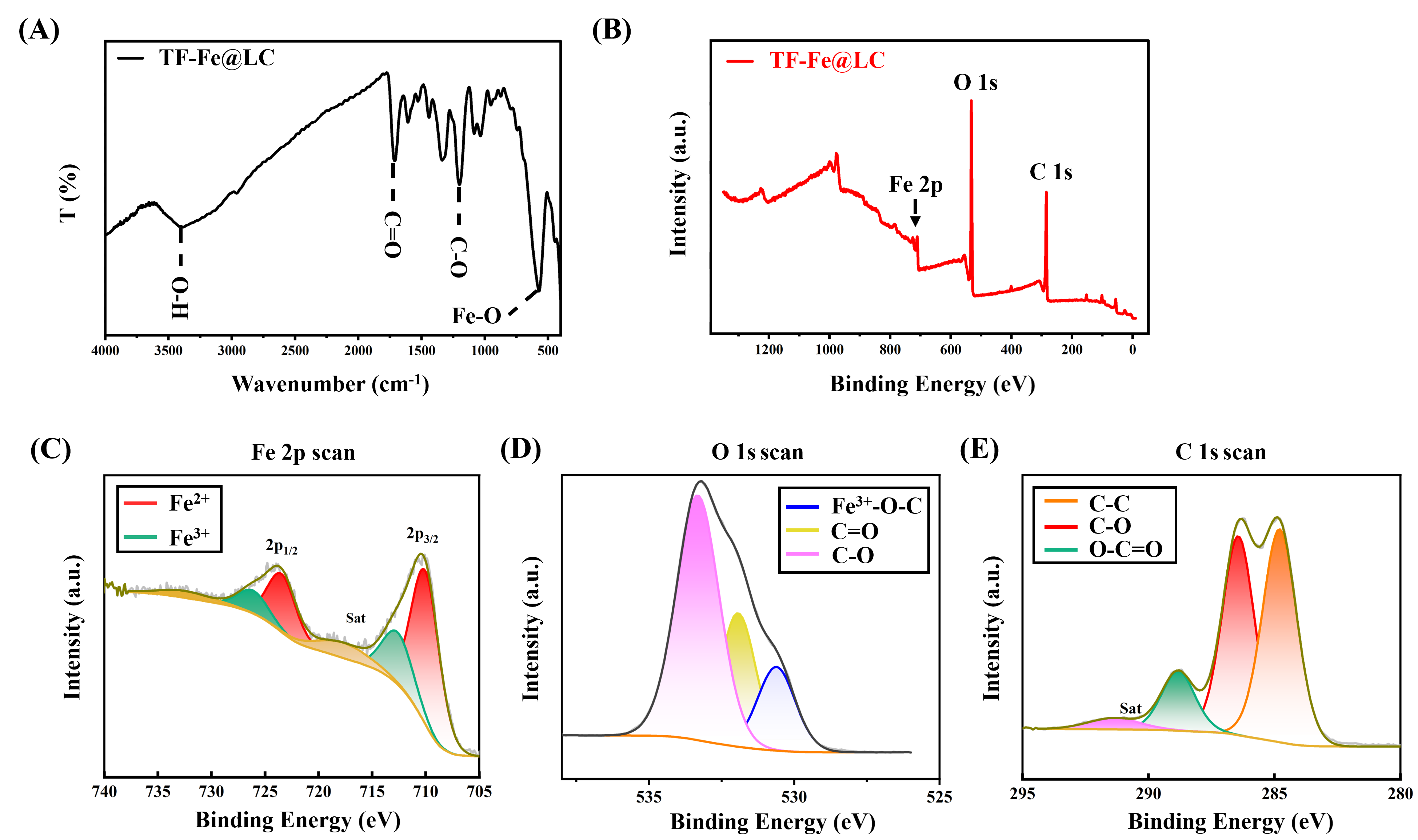


**Figure S5.** Characterization of TF-Fe@LC. (A) FT-IR of TF-Fe@LC. (B) XPS spectra of TF-Fe@LC. (C) High resolution XPS spectra of Fe in TF-Fe@LC. (D) High resolution XPS spectra of O in TF-Fe@LC. (E) High resolution XPS spectra of C in TF-Fe@LC.

**
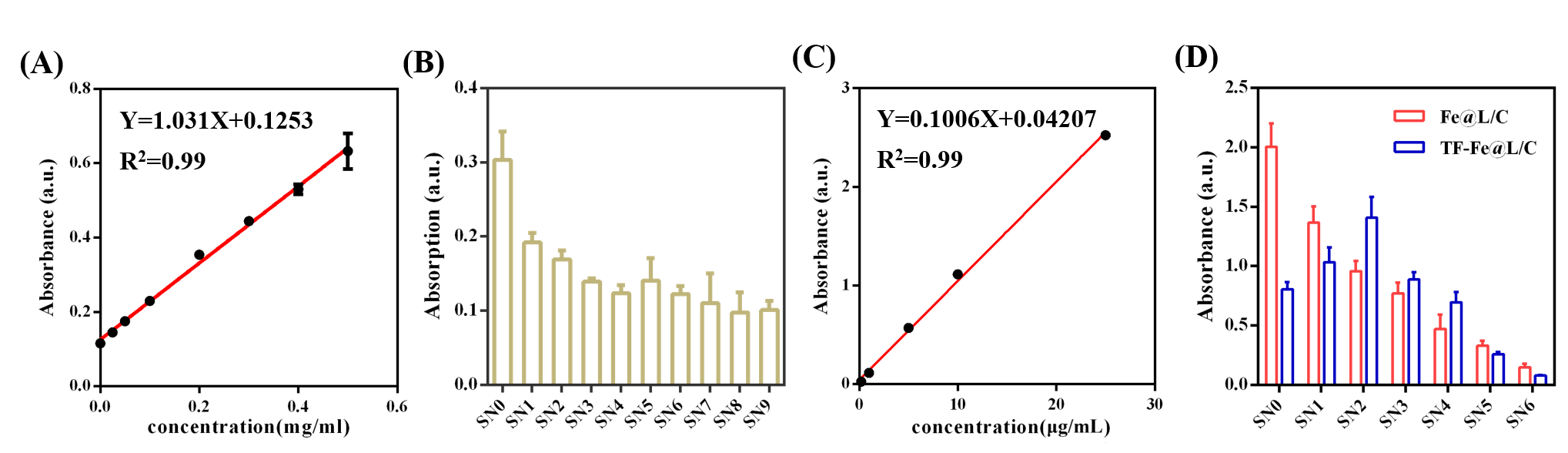
**

**Figure S6.** The loading efficiency of LOD and CCCP in TF-Fe@LC. (A) The standard curve of protein concentration versus absorbance. (B) Supernatant LOD concentration after each washing (n=3). (C) Standard curve of CCCP concentration versus absorbance. (D) Supernatant CCCP concentration after each washing (n=3).


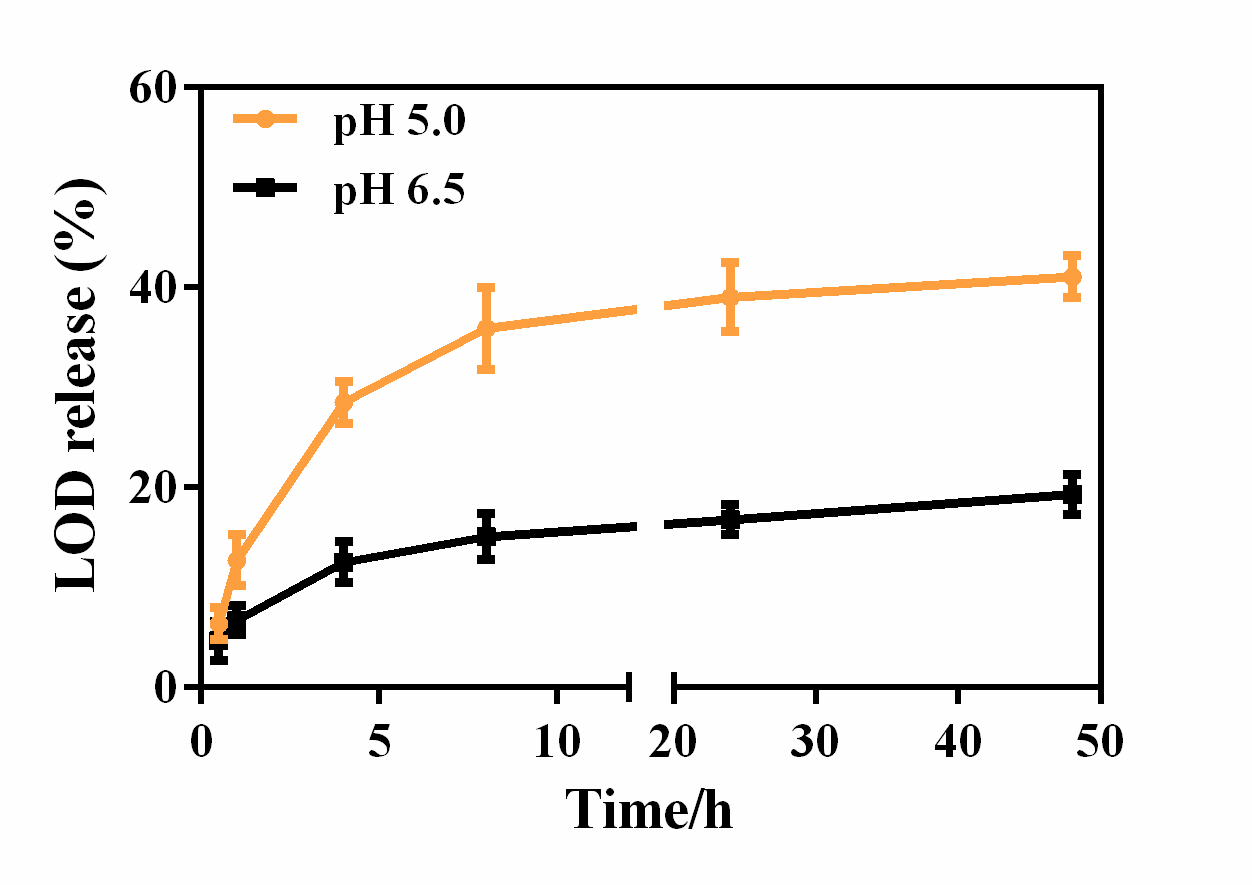


**Figure S7.** Time-course of LOD release from TF-Fe@LC at varying pH (n=3).

**
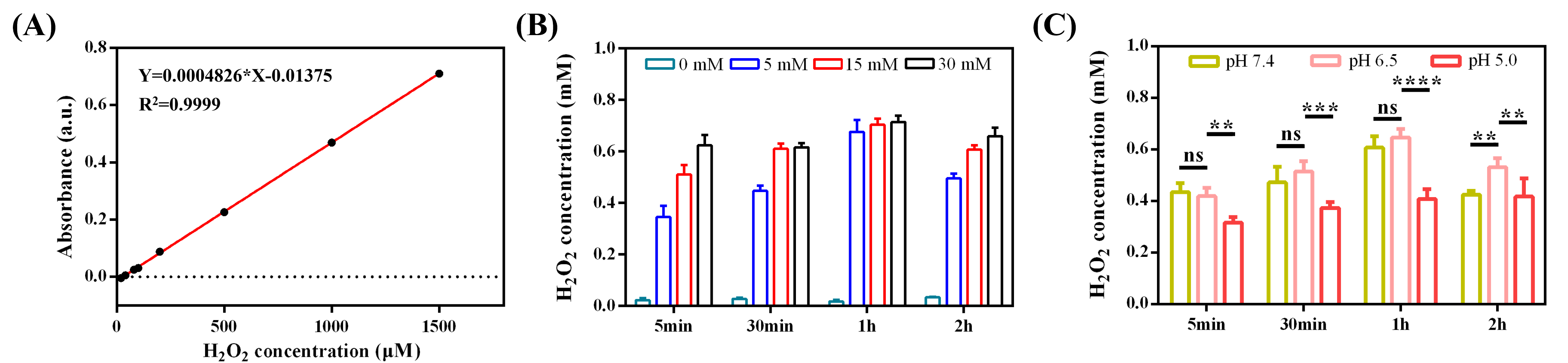
**

**Figure S8.** Detection of lactate oxidase-catalyzed H₂O₂ generation. (A) Standard curve for H₂O₂ concentration. (B) Time-course measurements of H₂O₂ generation in LOD solutions (4 U) supplemented with different NaL concentrations (n=3). (C) Time-course measurements of H₂O₂ generation in LOD solutions (4 U) in solutions containing 5 mM sodium lactate (NaL) at varying pH values (n=3). *^**^P*＜0.01, *^***^P*＜0.001, *^****^P*＜0.0001, ns: not significant.

**
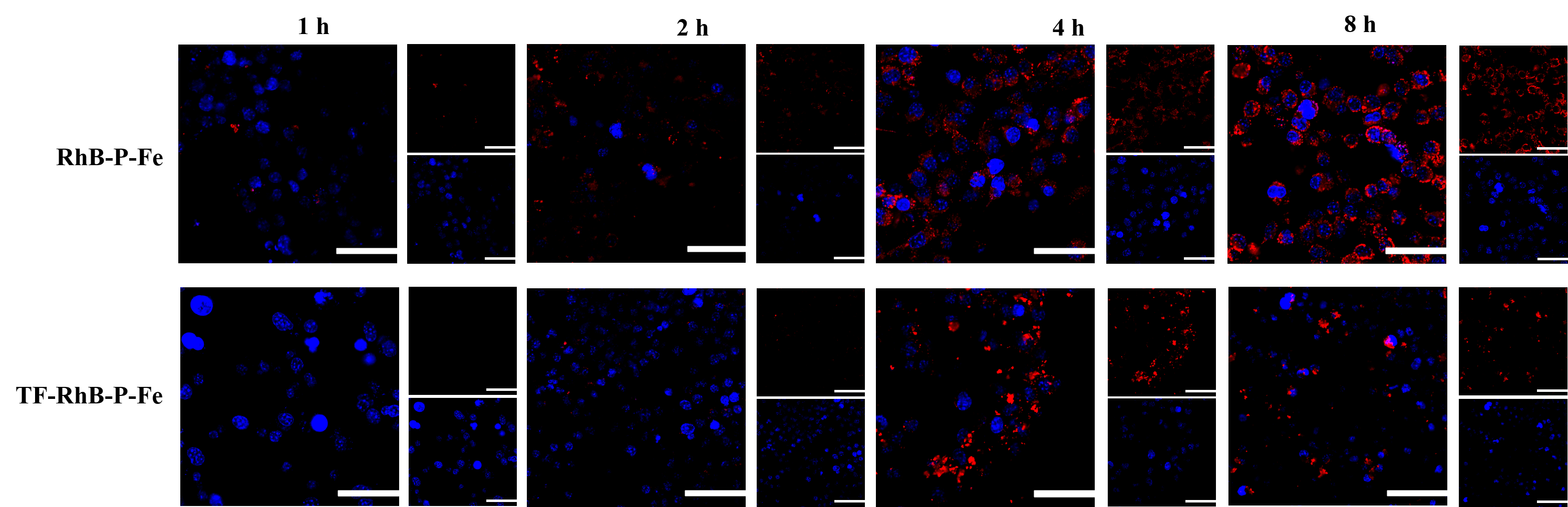
**

**Figure S9.** CLSM images of 4T1 cells treated with P-Fe or TF-Fe NCs for 1-8 h. Scale bar: 50 μm.

**
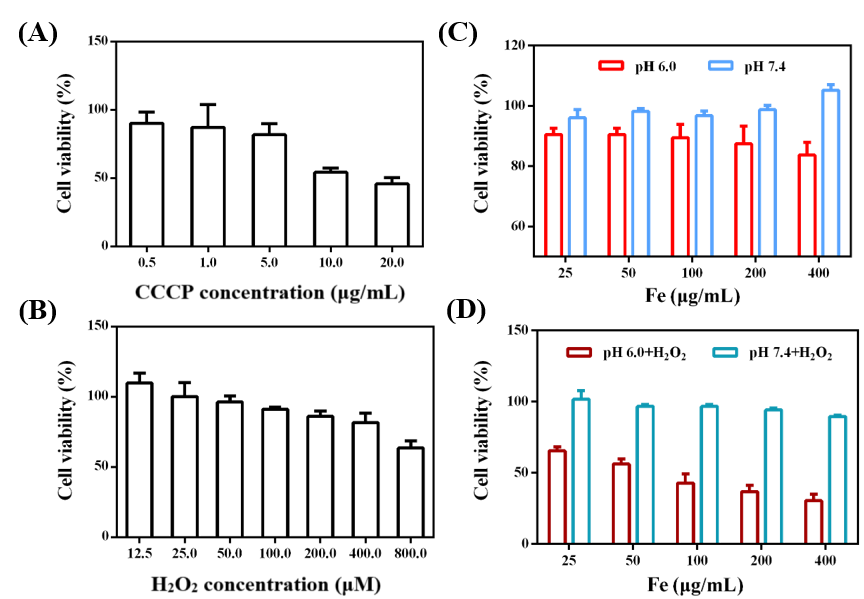
**

**Figure S10.** Cell viability of 4T1 cells with different treatments. (A) Various concentrations of CCCP. (B) Various concentrations of H_2_O_2_. (C) TF-Fe at varying concentrations under different pH conditions. (D) Various concentrations of TF-Fe with 100 μM of H_2_O_2_ under different pH conditions. The data were presented as the mean±SD (n = 6).


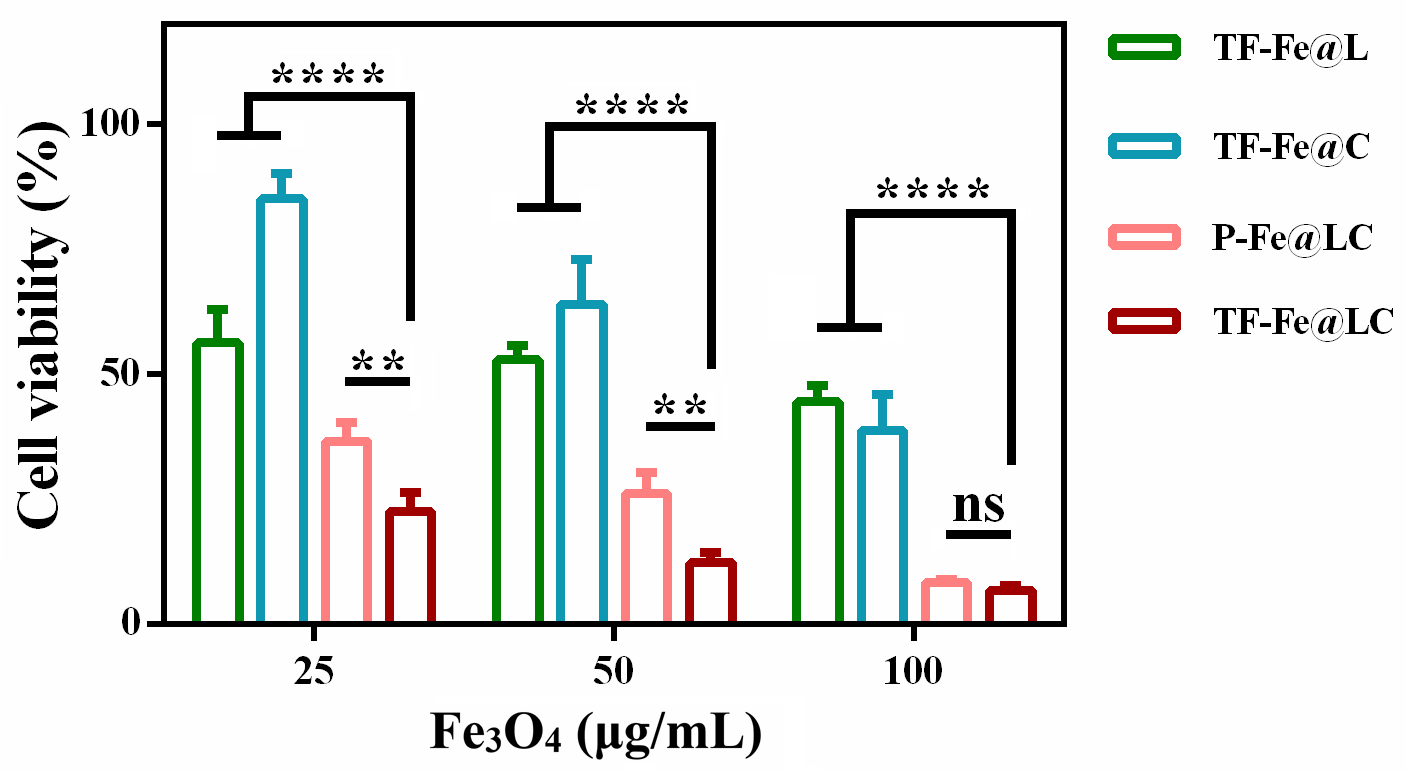


**Figure S11.** The effects of TF‑Fe@L, TF‑Fe@C, Fe@LC, and TF‑Fe@LC at different concentrations (25, 50, and 100 μg/mL) on the viability of 4T1 cells. n=6, *^**^P*＜0.01, *^****^P*＜0.0001, ns: not significant.


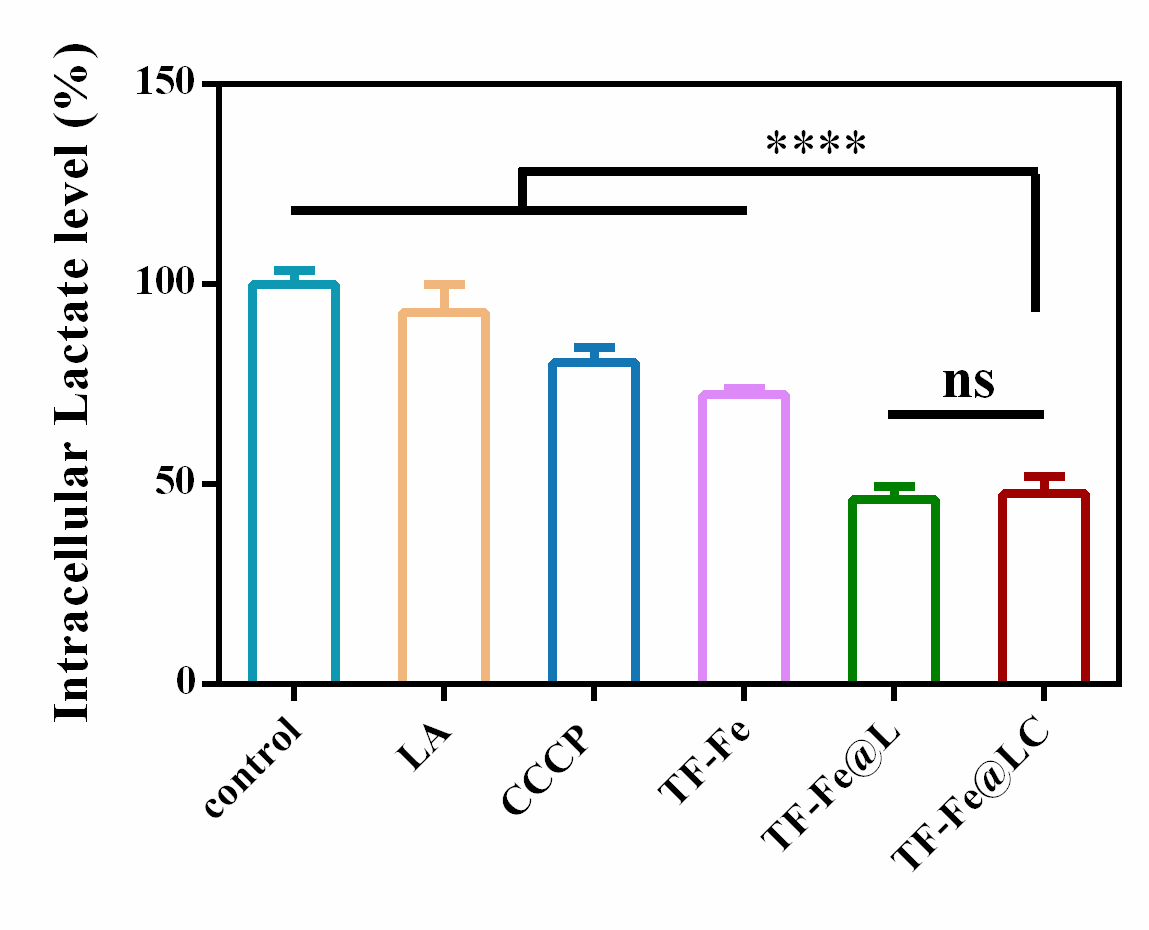


**Figure S12.** Intracellular lactate level in 4T1 cells upon different treatments for 12 h. The data were presented as the mean±SD, n = 3, ns: not significant, *^****^P* ˂ 0.0001.

**
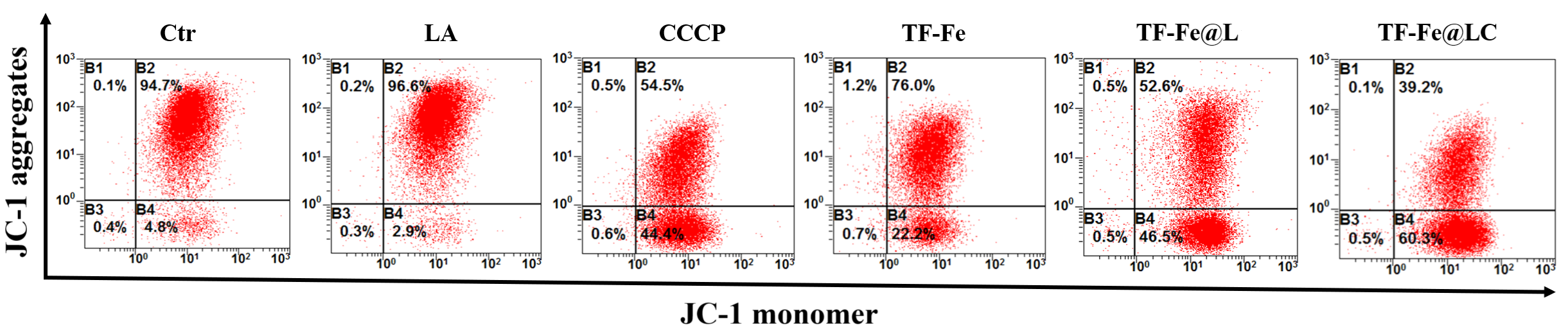
**

**Figure S13.** Representative flow cytometric analysis of MMP changes in 4T1 cells after different treatments.

**
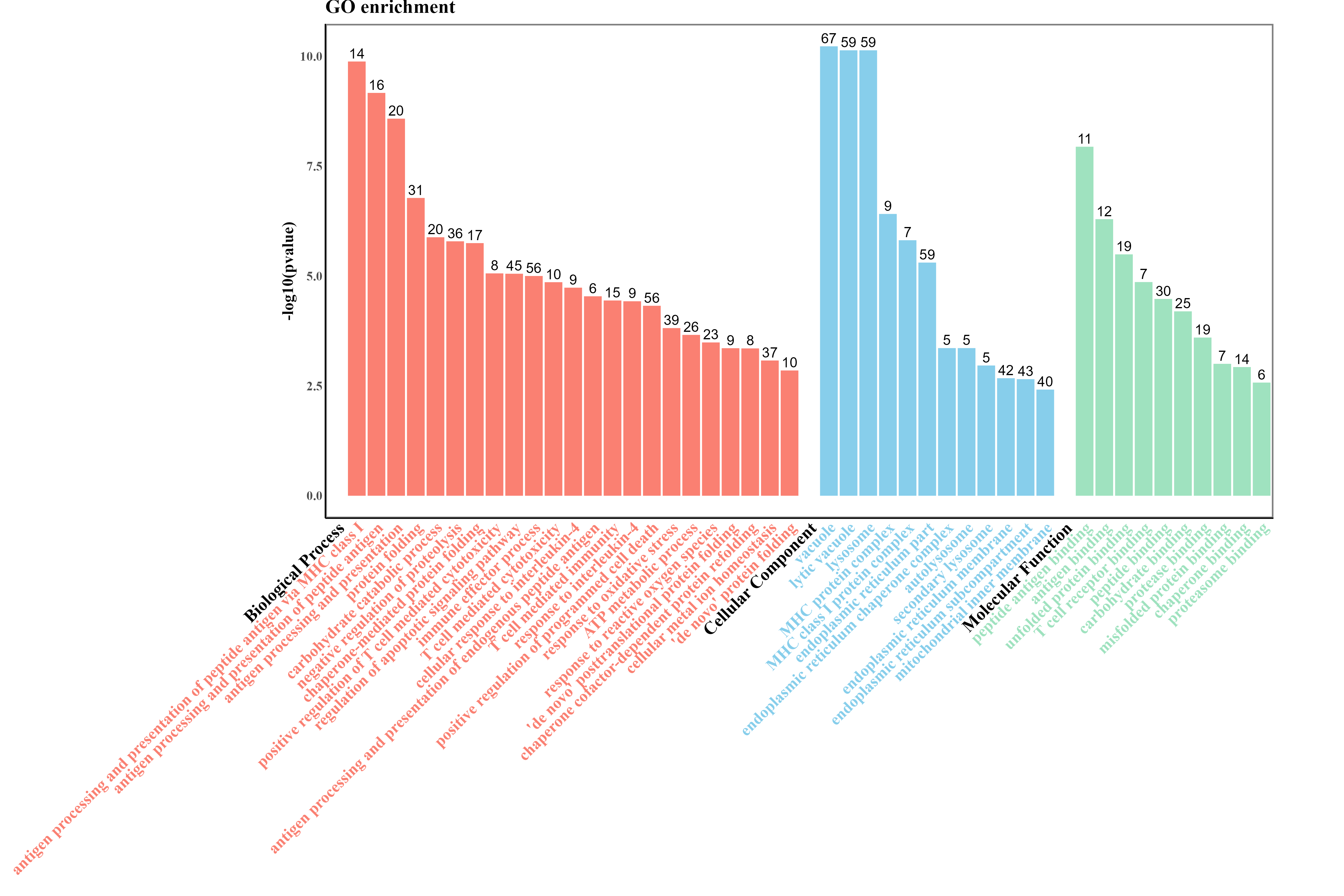
**

**Figure S14.** GO analysis of of the differentially expressed genes.

**
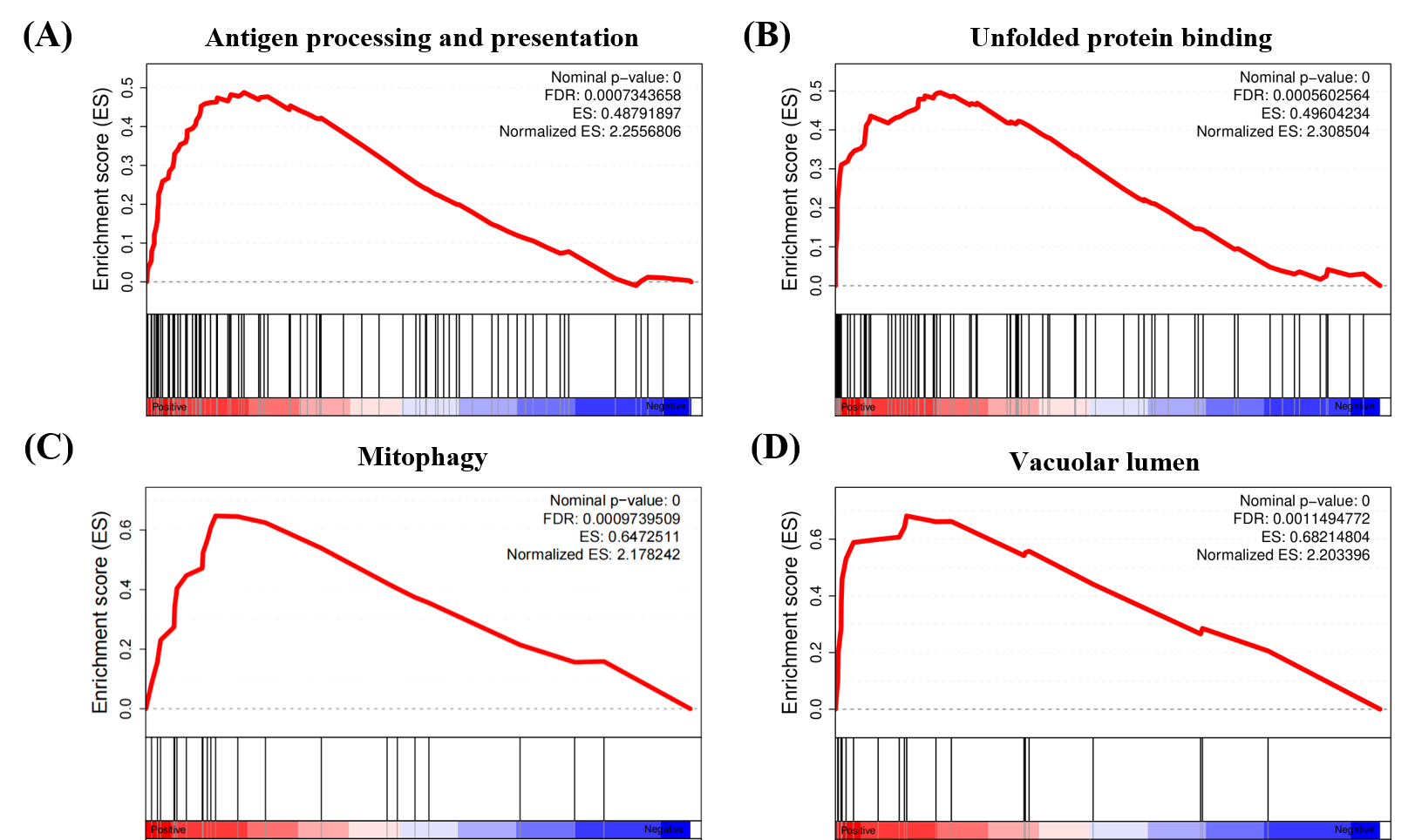
**

**Figure S15.** GSEA of differentially expressed genes, highlighting significant enrichment in key various cellular processes.


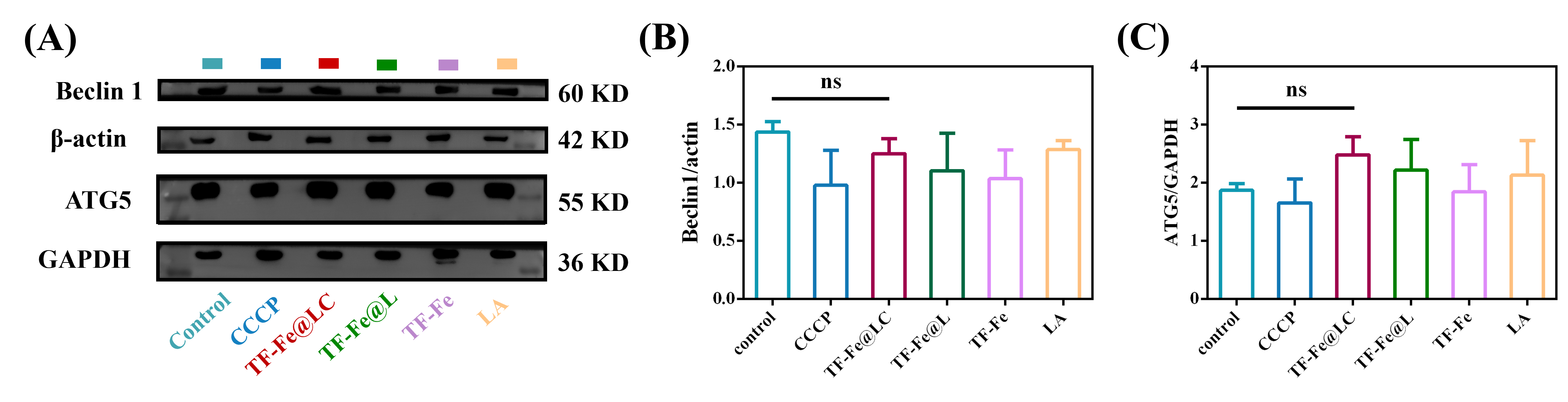


**Figure S16.** Western blot of autophagy initiation-related proteins in 4T1 cells under different treatments with corresponding proteins quantitative analysis. (A) The expression of Beclin1 and ATG5 proteins in 4T1 cells. The corresponding quantitative analysis of (B) Beclin1 and (C) ATG5. n=3, ns: not significant.

**
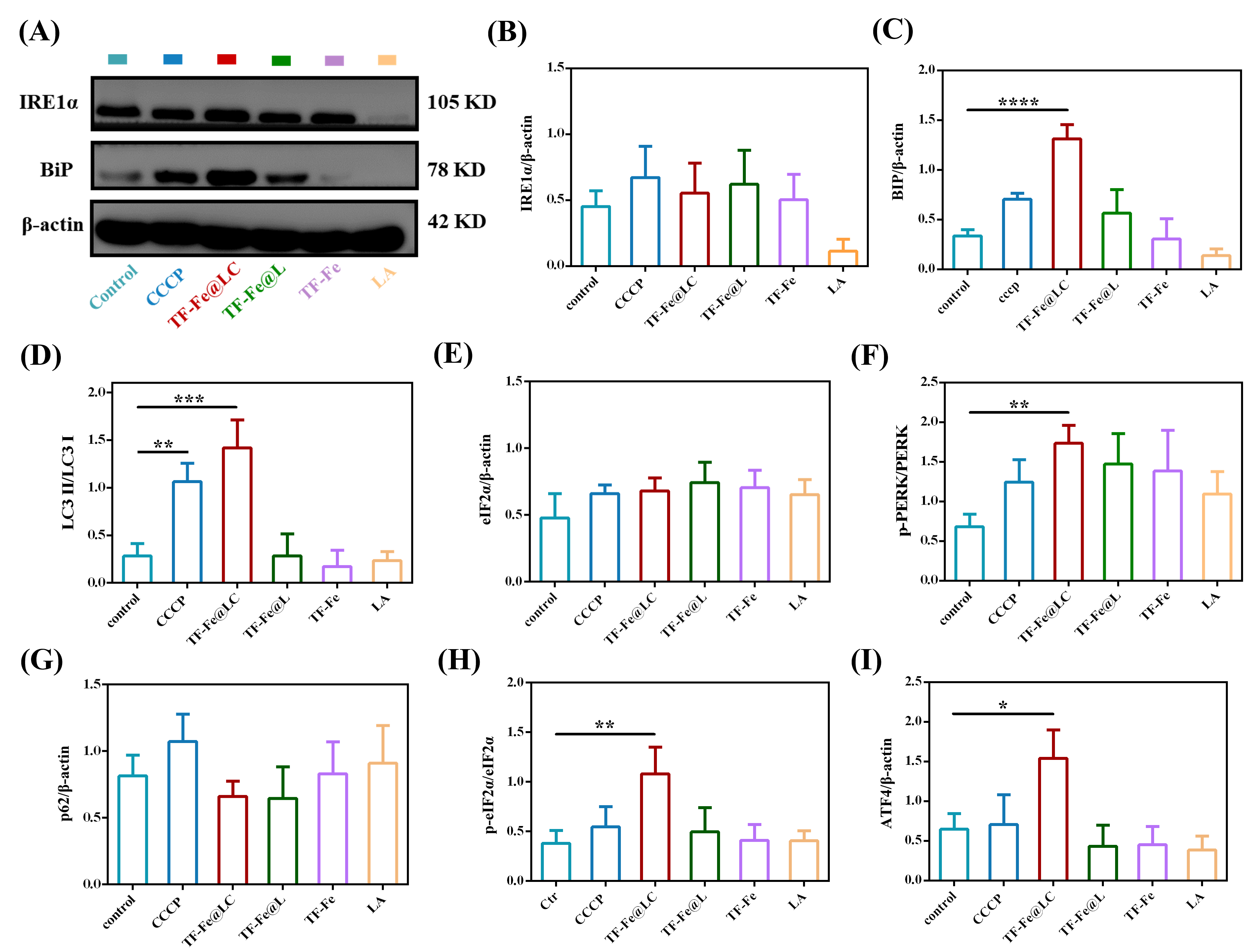
**

**Figure S17.** Western blot of ER stress-related proteins in 4T1 cells under different treatments with corresponding proteins quantitative analysis. (A) The expression of IRE1α and BiP proteins in 4T1 cells. The quantitative analysis of (B) IRE1α; (C) BiP; (D) LC3 II/LC3 I; (E) p62; (F) eIF2α; (G) p-eIF2α/eIF2α; (H) p-PERK/PERK; (I) ATF4. n=3, *^*^P* ˂ 0.05, *^**^P* ˂ 0.01, *^***^P* ˂ 0.001, *^****^P* ˂ 0.0001.

**
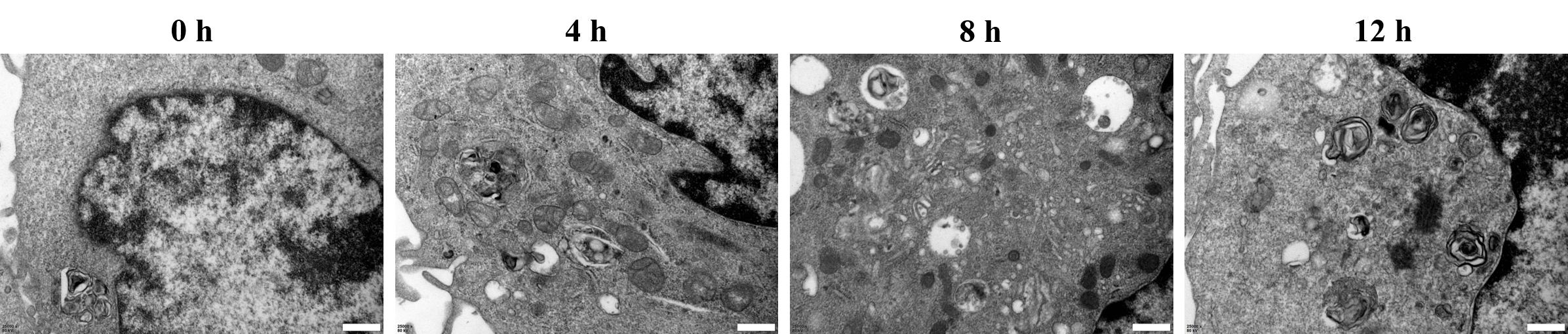
**

**Figure S18.** The observation of autophagy by Bio-TEM. Scale bar: 500 nm.


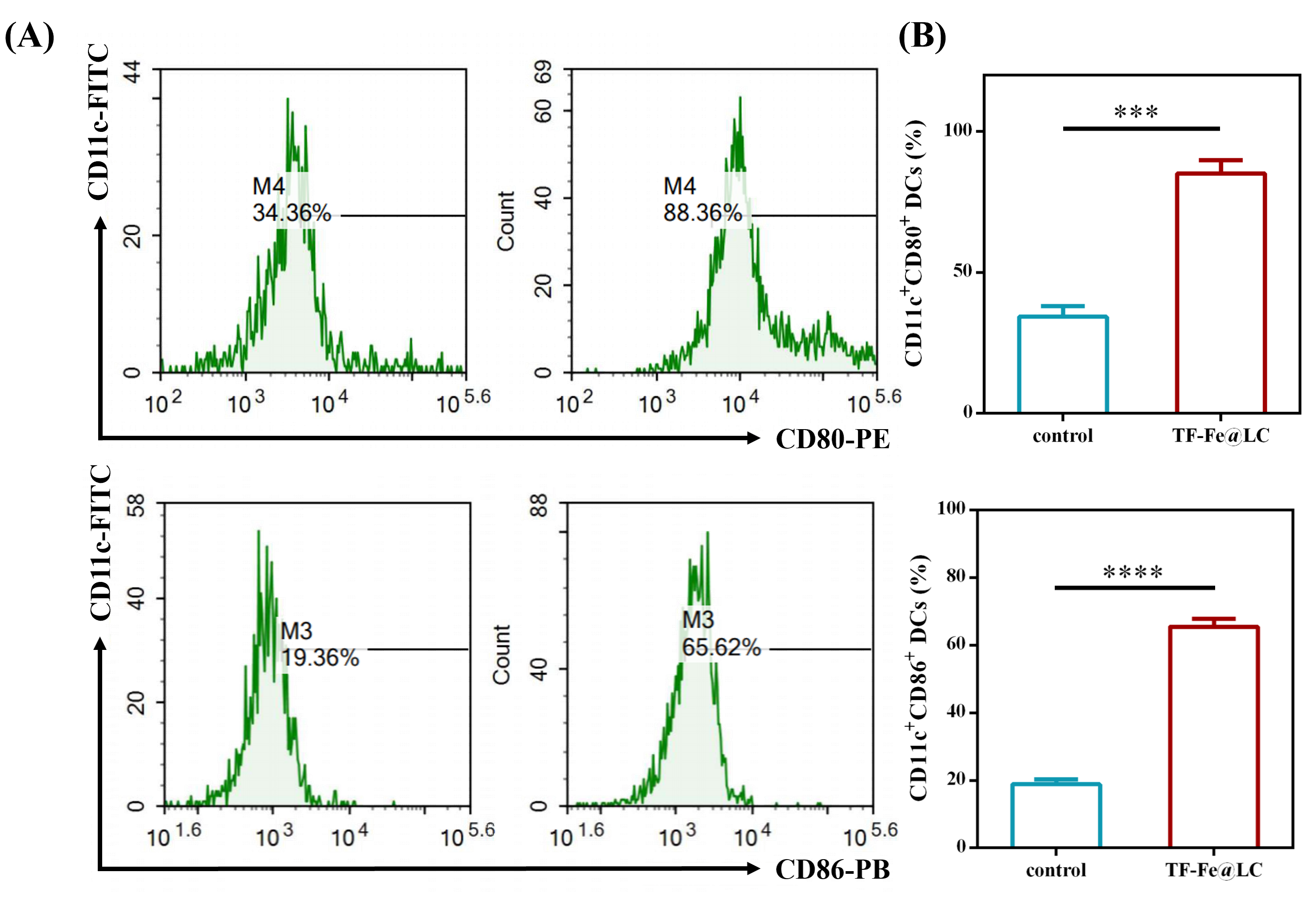


**Figure S19.** Analysis of DC maturation after co‑incubation with TF‑Fe@LC using flow cytometry. (A) Representative flow cytometry plots. (B) Corresponding quantitative analysis of DC maturation percentage. n=3, *^***^P* ˂ 0.001, *^****^P* ˂ 0.0001.

**
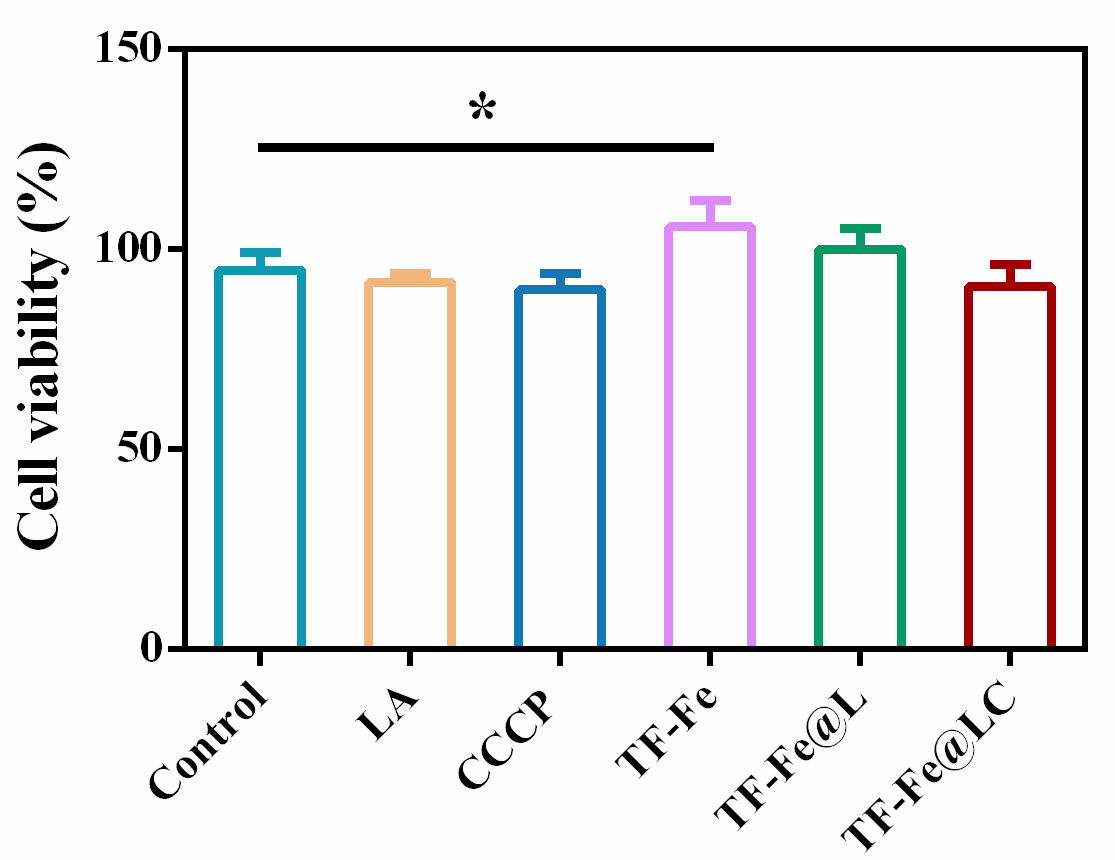
**

**Figure S20.** Cell viability of MCF10A cells with different treatments. The data were presented as the mean±SD, n = 6, *^*^P* ˂ 0.05.

**
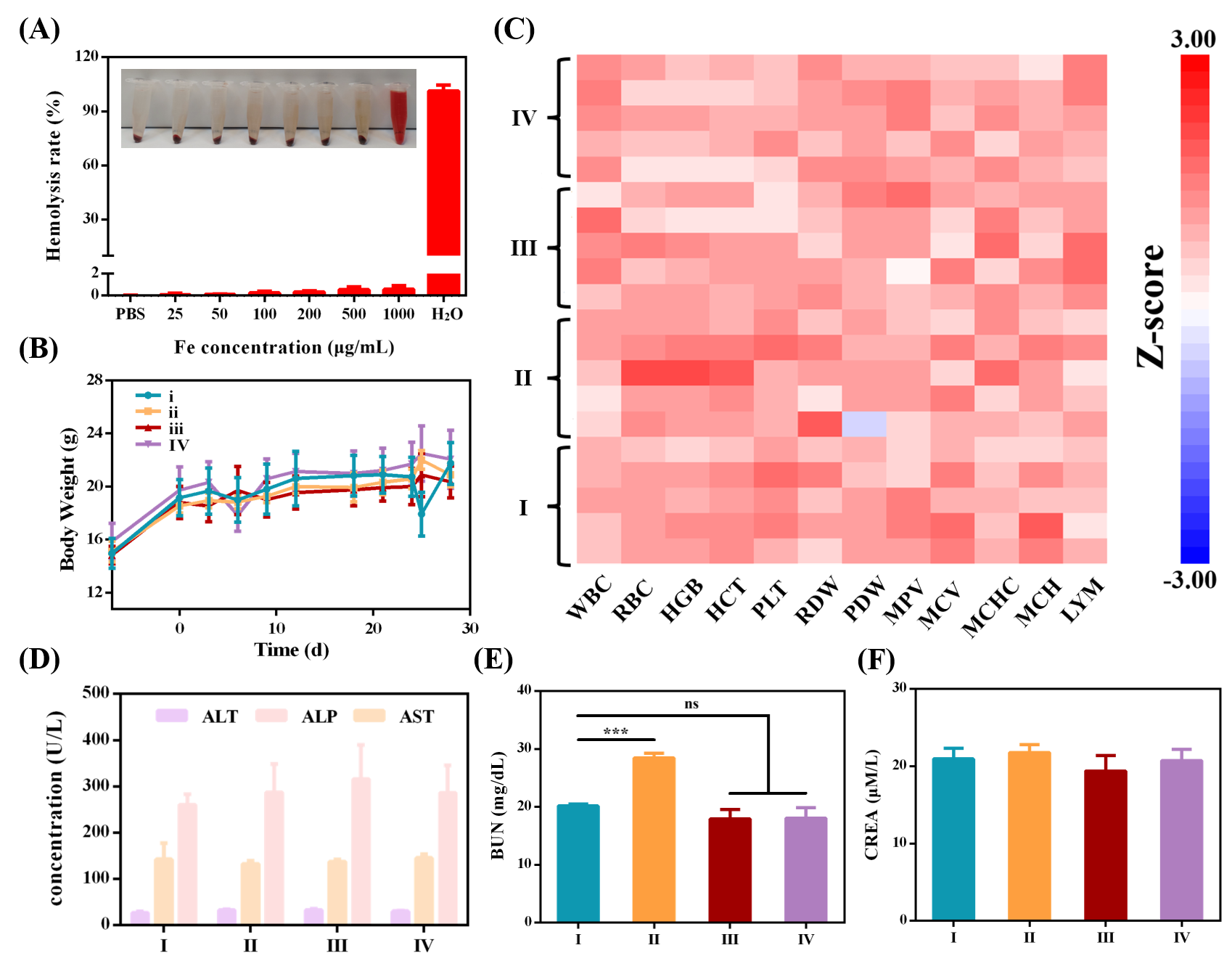
**

**Figure S21.** *In vitro and vivo* biosafety evaluation. (A) Hemolysis test (n=3). (B) Body weight changes of mice during the evaluation period. (C) Heatmap analysis of blood routine parameters in mice at observation termination. (D-F) Blood biochemical parameters of mice. All measurements were performed in mice (n=5/group) following treatment with varying doses of TF-Fe@LC. I, PBS; II, 10 mg/kg TF-Fe@LC; III, 20 mg/kg TF-Fe@LC; IV, 40 mg/kg TF-Fe@LC. *^**^**^*^P* ˂ 0.001, ns: not significant.

**
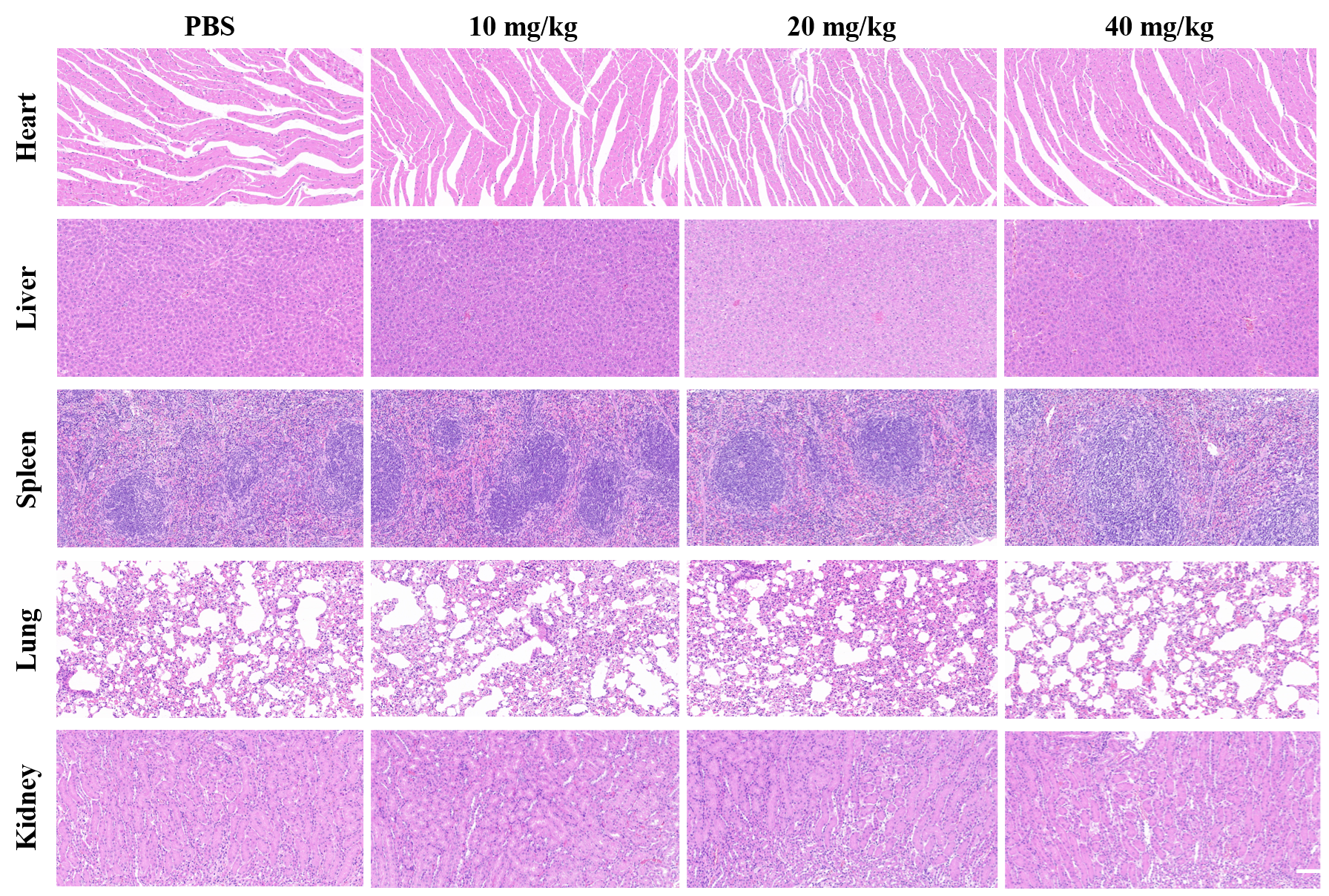
**

**Figure S22.** H&E staining of major organs (heart, liver, spleen, lung, kidney) in mice after treatment with different doses of TF-Fe@LC. Scale bar: 100 nm.

**
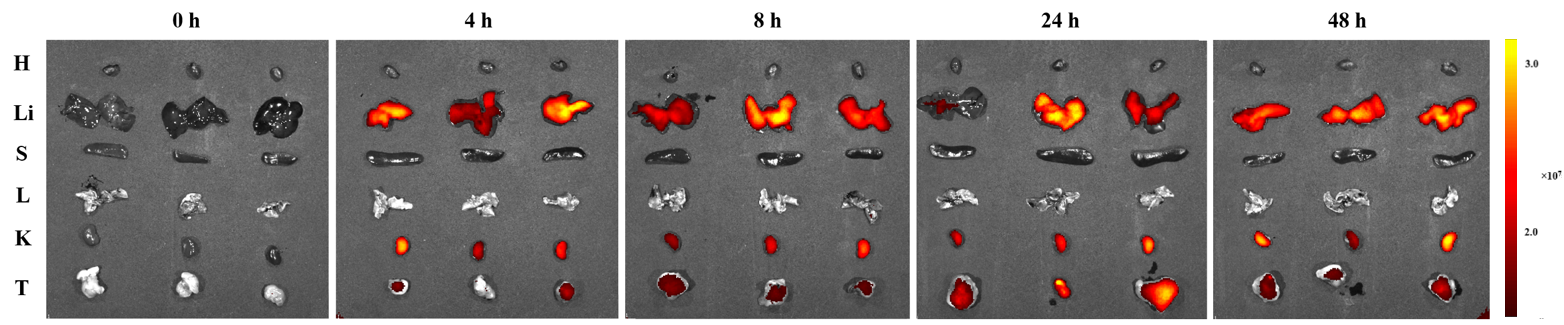
**

**Figure S23.** Representative *ex vivo* fluorescence images of RhB-labeled TF-Fe@LC distribution in tumors and organs (heart, liver, spleen, lung, kidney) at various time points post-injection (n=5).


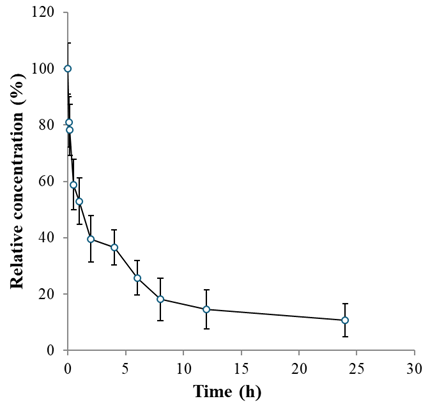


**Figure S24.** Plasma concentration-time curve of RhB labeled TF-Fe@LC after intravenous injection. n=3

**
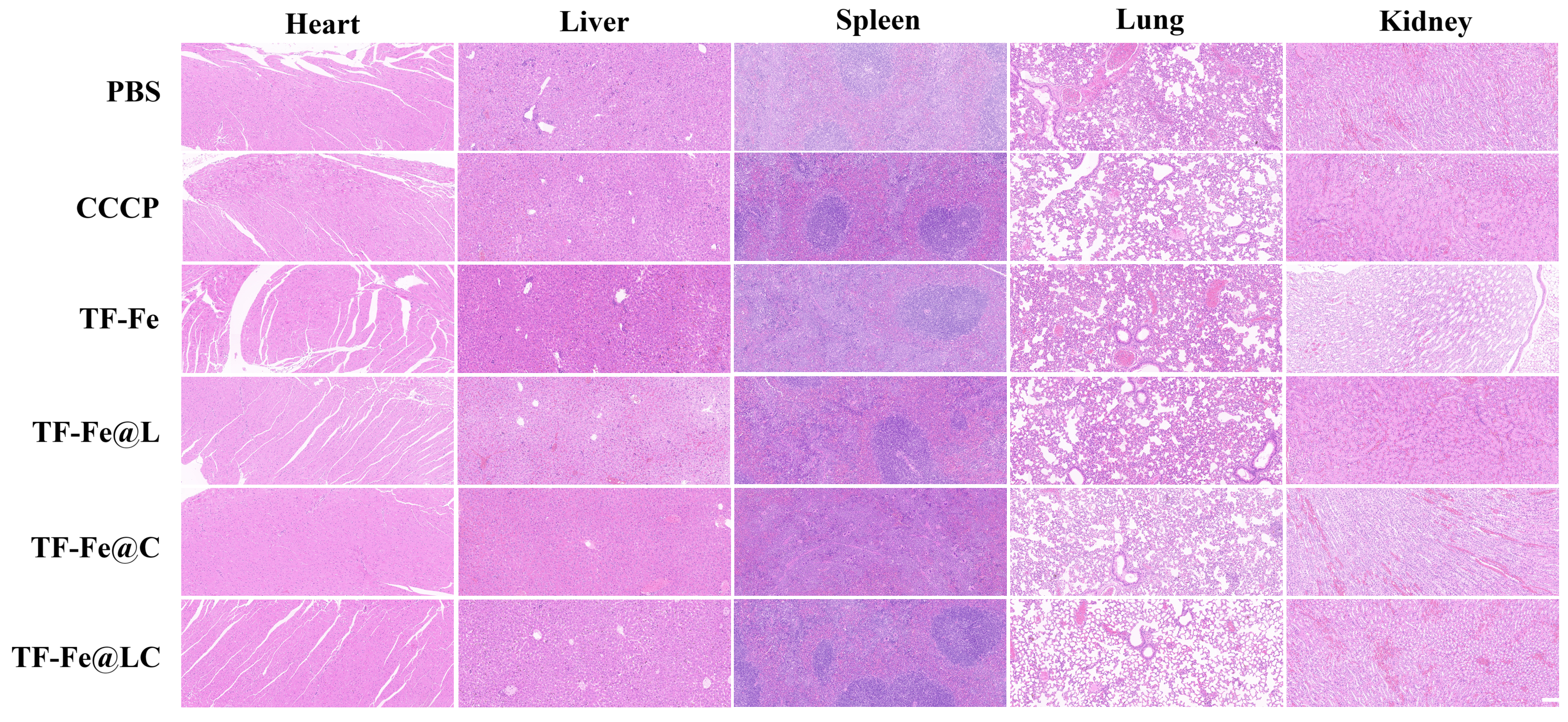
**

**Figure S25.** H&E staining of major organs from mice following different treatments. Scale bar: 100 nm.

**
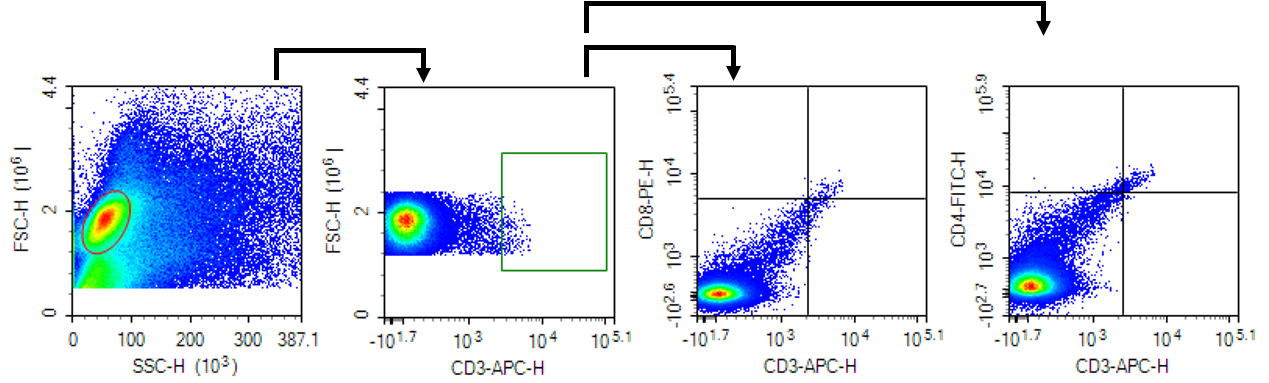
**

**Figure S26.** Flow Cytometry Gating Strategy for splenic CD3^+^CD8^+^ and CD3^+^CD4^+^ T cells.

**
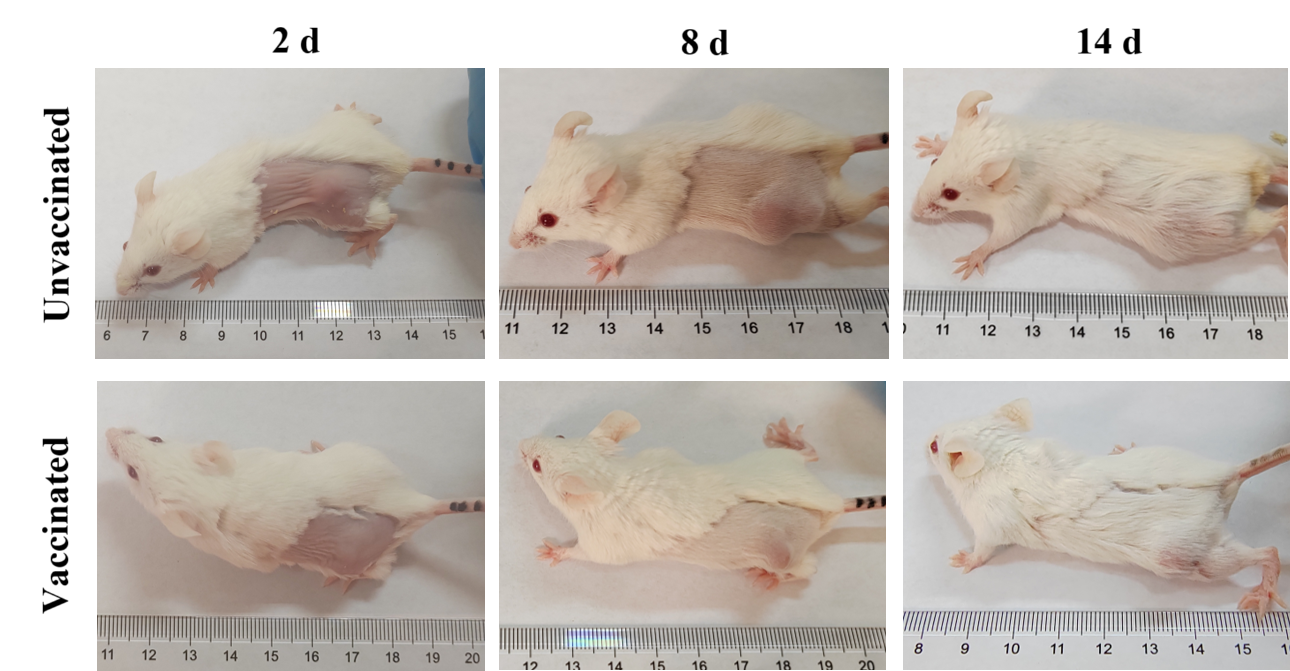
**

**Figure S27.** Representative photographs of unvaccinated/vaccinated mice captured at defined timepoints.

**
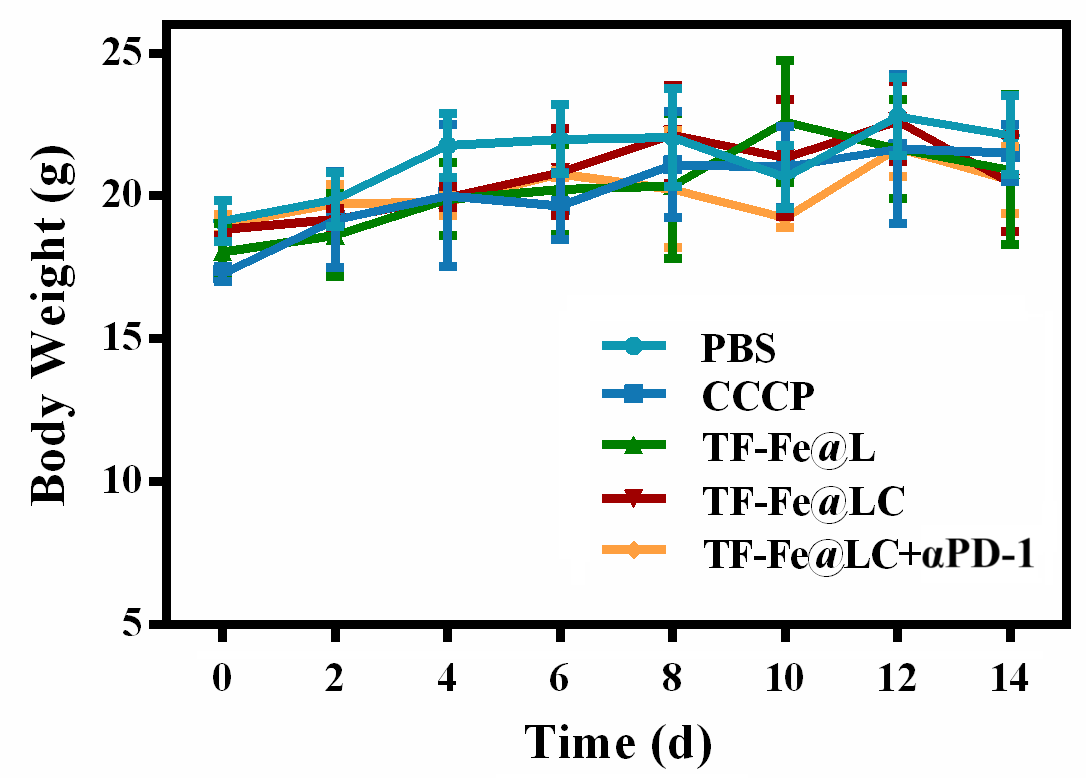
**

**Figure S28.** Body weight of mice in bilateral tumor model (n=5).

**
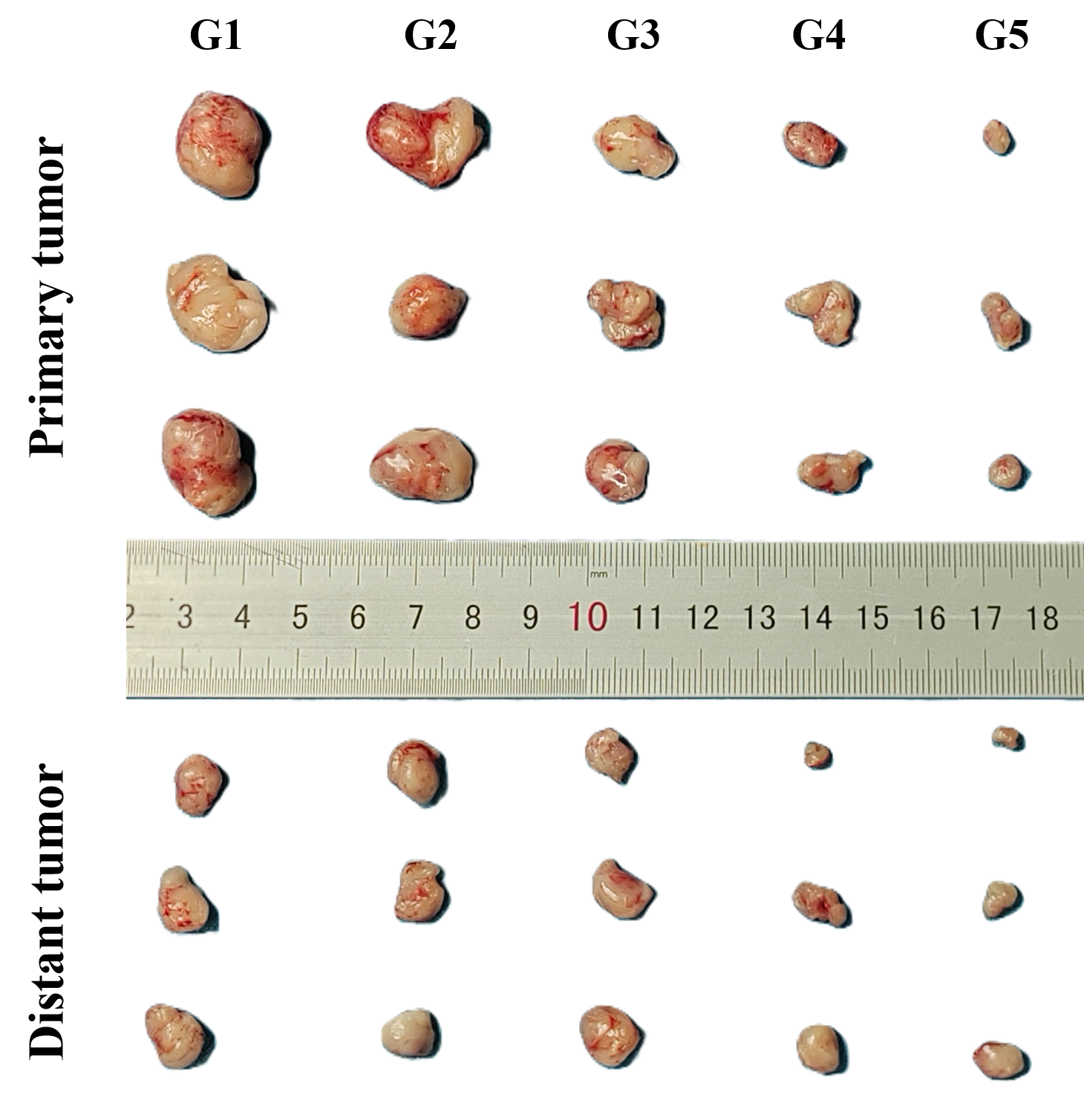
**

**Figure S29.** Photographs of the excised tumors in bilateral tumor model (G1: PBS, G2: CCCP, G3: TF-Fe@L, G4: TF-Fe@LC, G5: TF-Fe@LC+αPD-L1; n=3).

**
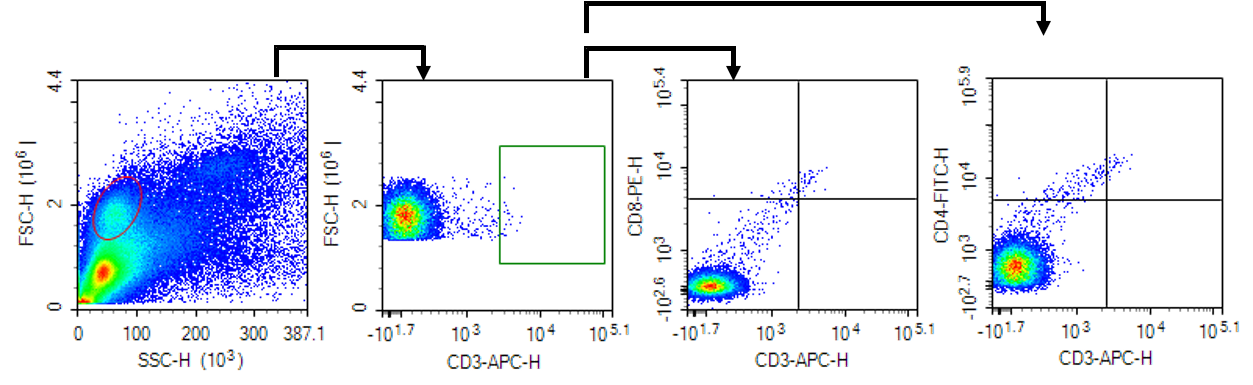
**

**Figure S30.** Flow Cytometry Gating Strategy for tumor-infiltrating CD8^+^ and CD4^+^ T cells.

**
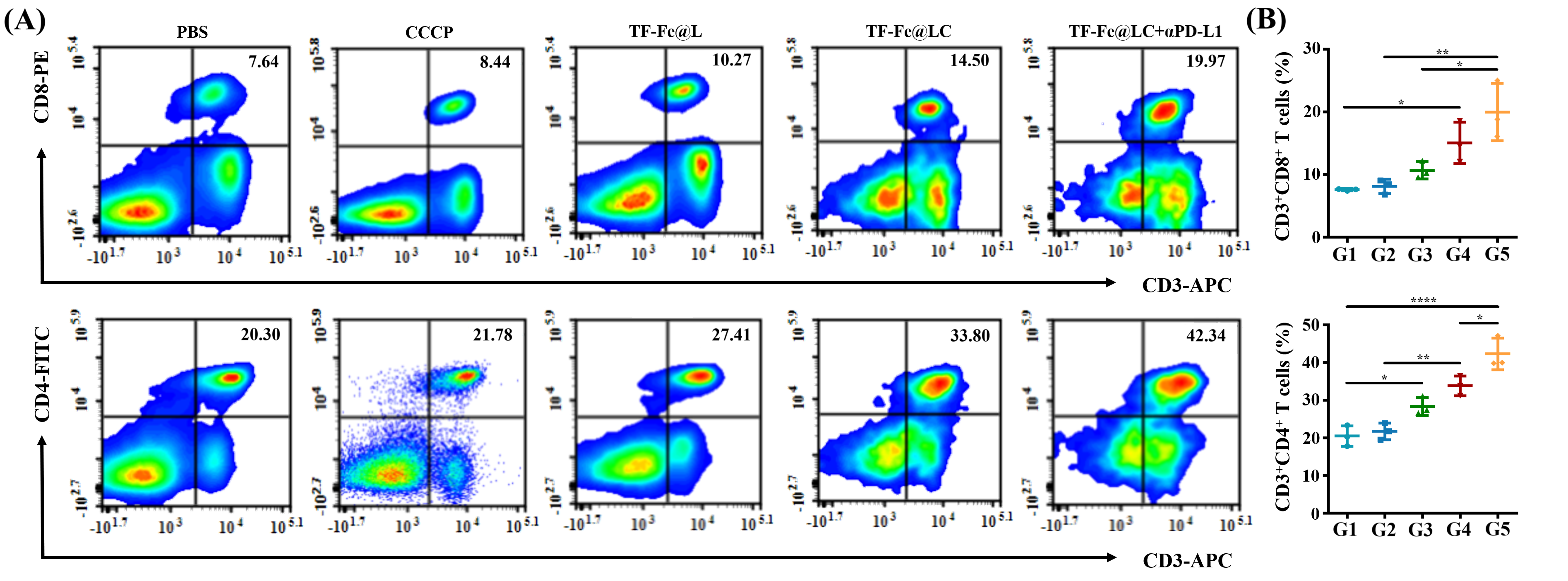
**

**Figure S31.** (A) Representative flow cytometric plots and (B) quantitative analysis of CD4⁺ (CD3⁺CD4⁺) and CD8⁺ (CD3⁺CD8⁺) T cells in primary tumors after different treatments (G1: PBS, G2: CCCP, G3: TF-Fe@L, G4: TF-Fe@LC, G5: TF-Fe@LC+αPD-L1). n=3, *^*^P* ˂ 0.05, *^**^P* ˂ 0.01, *^****^P* ˂ 0.0001.


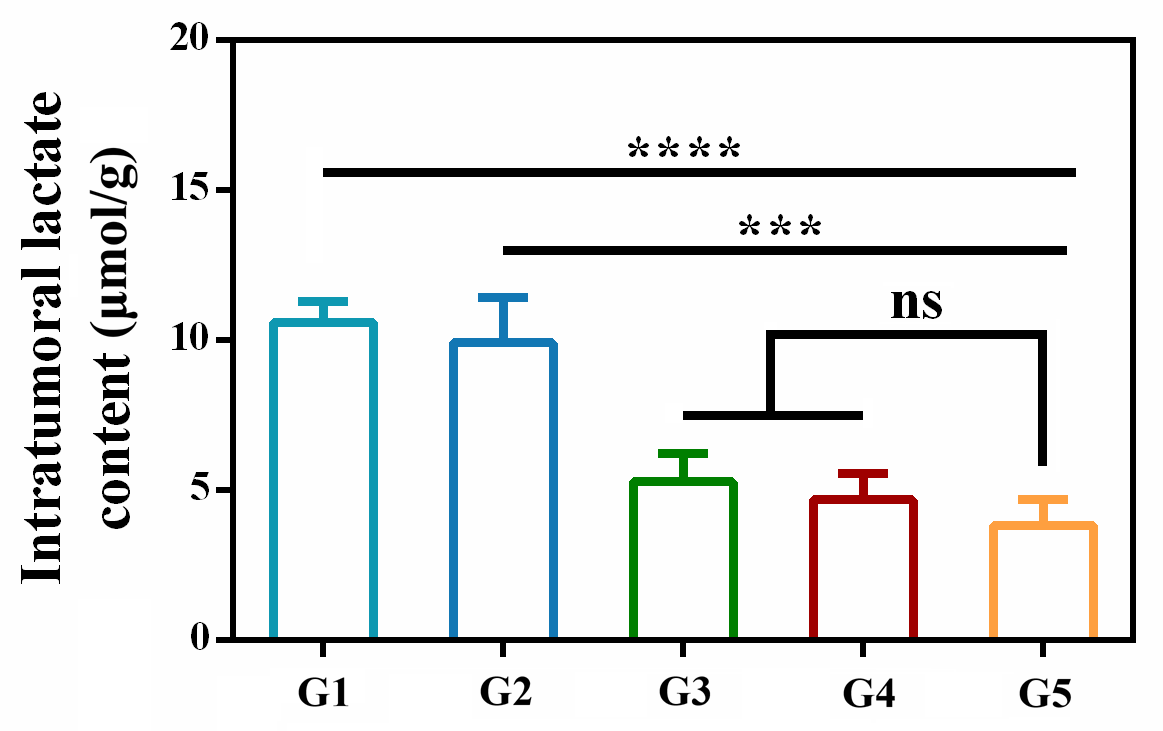


**Figure S32.** The intratumoral lactate content in abscopal tumors 24 h post-treatment (G1: PBS, G2: CCCP, G3: TF-Fe@L, G4: TF-Fe@LC, G5: TF-Fe@LC+αPD-L1). n=3, *^***^P* ˂ 0.001, ns: not significant.

**
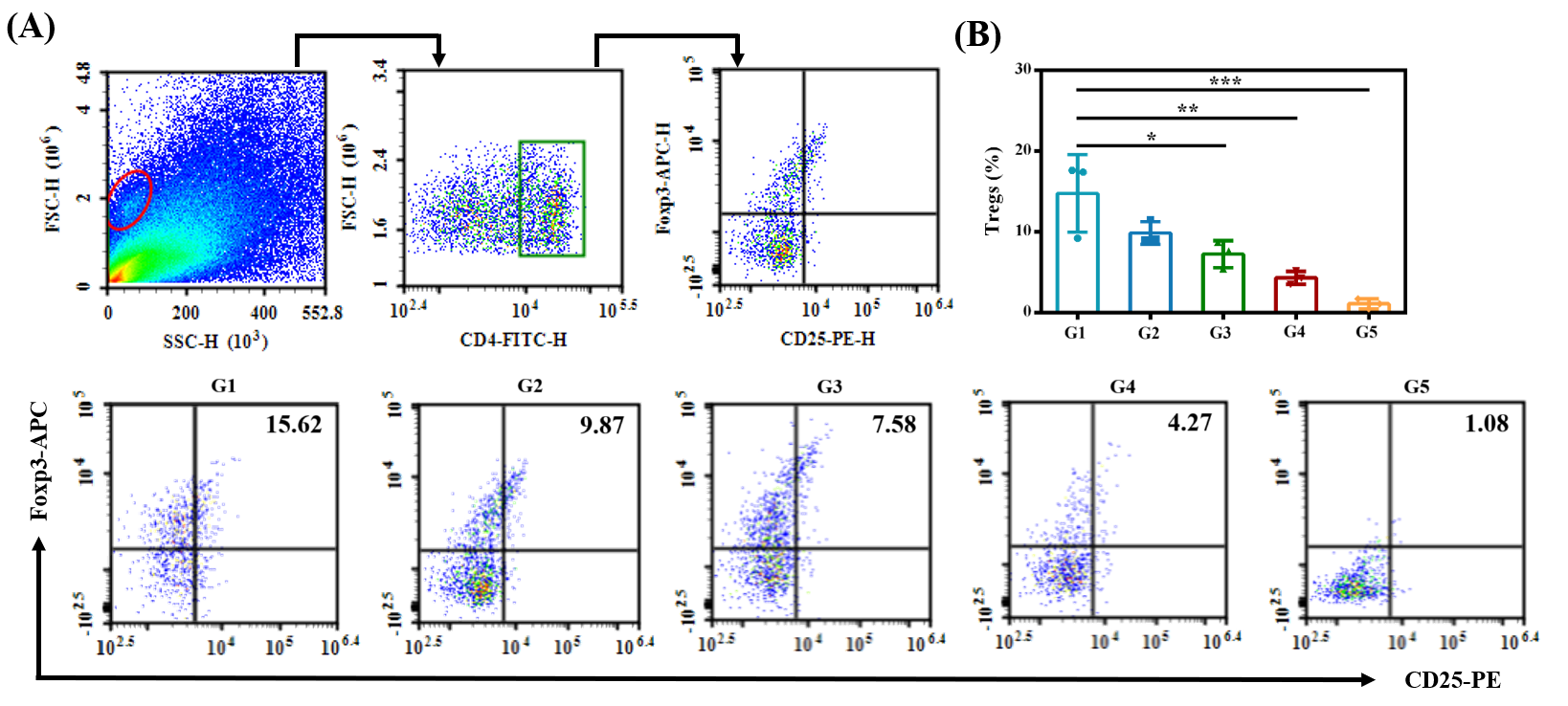
**

**Figure S33.** (A) Flow Cytometry Gating Strategy for Tregs and representative plots depicting Tregs populations in distant tumors. (B) Quantification of Treg percentages in distant tumors across different treatments (G1: PBS, G2: CCCP, G3: TF-Fe@L, G4: TF-Fe@LC, G5: TF-Fe@LC+αPD-L1). n=3, *^*^P* ˂ 0.05, *^**^P* ˂ 0.01, *^***^P* ˂ 0.001.

**
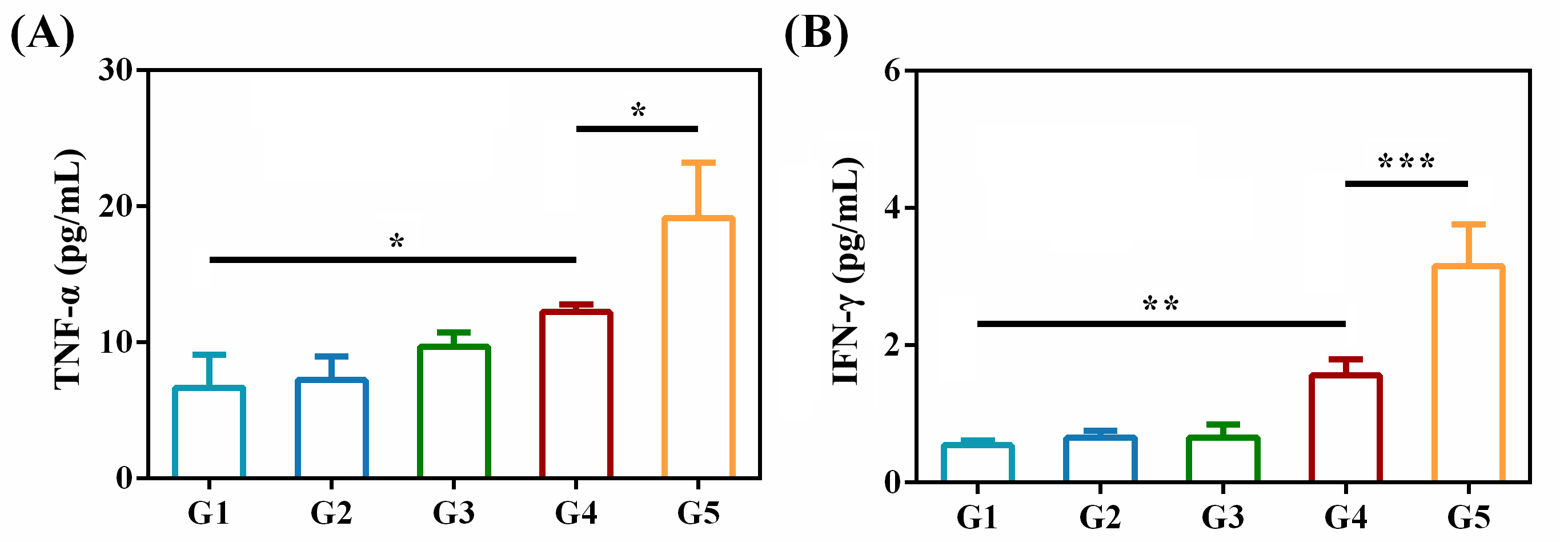
**

**Figure S34.** ELISA detection of the (A) TNF-α and (B) IFN-γ in the serum of 4T1 bilateral tumor-bearing mice across the treatment cohorts (G1: PBS, G2: CCCP, G3: TF-Fe@L, G4: TF-Fe@LC, G5: TF-Fe@LC+αPD-L1). n=3, *^*^P* ˂ 0.05, *^**^P* ˂ 0.01, *^***^P* ˂ 0.001, ns: not significant.

**References**

[1] C. Zhang et al., Colloids Surf., B. 229 (2023) 113467.

[2] K. Cheng et al., J. Am. Chem. Soc. 131 (30) (2009).
